# Microalgae as next generation plant growth additives: Functions, applications, challenges and circular bioeconomy based solutions

**DOI:** 10.3389/fpls.2023.1073546

**Published:** 2023-03-30

**Authors:** Priyanka Parmar, Raman Kumar, Yograj Neha, Vidyashankar Srivatsan

**Affiliations:** ^1^ Applied Phycology and Food Technology Laboratory, Council of Scientific and Industrial Research (CSIR)- Institute of Himalayan Bioresource Technology, Palampur, Himachal Pradesh, India; ^2^ Academy of Scientific and Innovative Research (AcSIR), Council of Scientific and Industrial Research -Human Resource Development Centre (CSIR-HRDC), Ghaziabad, Uttar Pradesh, India

**Keywords:** biostimulant, biofertilizers, biopesticides, biorefinery, circular economy, stress tolerance, bioremediation

## Abstract

Sustainable agriculture practices involve the application of environment-friendly plant growth promoters and additives that do not negatively impact the health of the ecosystem. Stringent regulatory frameworks restricting the use of synthetic agrochemicals and the increase in demand for organically grown crops have paved the way for the development of novel bio-based plant growth promoters. In this context, microalgae biomass and derived agrochemicals offer novel sources of plant growth promotors that enhance crop productivity and impart disease resistance. These beneficial effects could be attributed to the presence of wide range of biomolecules such as soluble amino acid (AA), micronutrients, polysaccharides, phytohormones and other signaling molecules in microalgae biomass. In addition, their phototrophic nature, high photosynthetic efficiency, and wide environmental adaptability make them an attractive source of biostimulants, biofertilizers and biopesticides. The present review aims to describe the various plant growth promoting metabolites produced by microalgae and their effects on plant growth and productivity. Further, the effects elicited by microalgae biostimulants with respect to different modes of applications such as seed treatments, foliar spray and soil/root drenching is reviewed in detail. In addition, the ability of microalgae metabolites to impart tolerance against various abiotic and biotic stressors along with the mechanism of action is discussed in this paper. Although the use of microalgae based biofertilizers and biostimulants is gaining popularity, the high nutrient and water requirements and energy intensive downstream processes makes microalgae based technology commercially unsustainable. Addressing this challenge, we propose a circular economy model of microalgae mediated bioremediation coupled with biorefinery approaches of generating high value metabolites along with biofertilizer applications. We discuss and review new trends in enhancing the sustainability of microalgae biomass production by co-cultivation of algae with hydroponics and utilization of agriculture effluents.

## Graphical Abstract

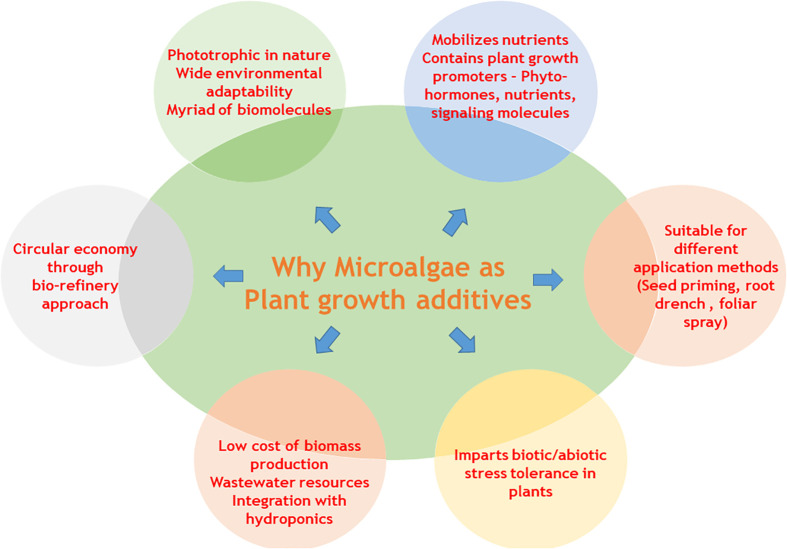

## 1 Introduction

The global population is projected to reach 9.6 billion by 2050 and the demand for food production is continuously increasing (Wu et al., 2014). However, the arable land available for crop production is limited and is expected to grow at a very negligible rate of 0.10% from 1592 million ha in 2005-07 estimate to a projected 1661 million ha in 2050 (Alexandratos and Bruinsma, 2012). To meet the global food requirements, intensive agricultural practices have been followed such as the use of chemical fertilizers, pesticides, and growth enhancers for maximizing crop productivity. Continuous use of these chemicals has deteriorated soil health mainly the physicochemical profile and soil micro-flora reducing the crop yield (Abinandan et al., 2019). This has led to a range of environmental issues such as nutrient leaching, contamination of surface and groundwater, eutrophication, greenhouse gas emissions, loss of aquatic biodiversity, and xenobiotics-induced human diseases (Mahapatra et al., 2022). In addition to the environmental concerns, the depletion of fossil fuels and non-renewable resources makes synthetic/chemical-based agriculture expensive (Woods et al., 2010). Further, the increasing consumer demand for organically grown crops and pesticide-free agriculture commodities necessitates the identification of safe, biologically derived, and sustainable alternatives for agricultural applications.

According to the European Biostimulant Industry Council (EBIC, 2022, https://biostimulants.eu/highlights/economic-overview-of-the-european-biostimulants-market/), a plant biostimulant refers to a material or a formulation which contains substance(s) and/or microorganisms whose function, when applied to plants or the rhizosphere is to stimulate natural processes to benefit nutrient uptake, nutrient efficiency, tolerance to abiotic stress, and/or crop quality, independently of its nutrient content (Ricci et al., 2019). In this context, microalgae and cyanobacteria have the potential to act as environmental friendly biostimulants/biofertilizers that improve crop quality and yield (Gonçalves, 2021). Microalgae are unicellular, mostly phototrophic organisms with wide environmental adaptability (Barsanti and Gualtieri, 2018). The ability of microalgae biomass to elicit a positive impact on plant growth and soil health could be attributed to the presence of a wide range of biomolecules such as N-fixing enzymes, soluble AAs, bio-mineral conjugates, polysaccharides and phytohormones (Kapoore et al., 2021; Lee and Ryu, 2021). Microalgae have been projected as a potential industrial feedstock owing to their high photosynthetic efficiency, their ability to grow in non-potable waters such as industrial effluents, and their ability to modulate metabolite biosynthetic pathways in response to varying environment (Ahmed et al., 2022; Chen et al., 2022; Zhao et al., 2022). Some of the industrially important microalgae species such as *Arthrospira platensis* (*Spirulina* spp.), *Chlorella* spp.*, Heamatococcus pluvialis, Dunaliella salina, Nostoc* spp.*, Anabaena* spp., *Scenedesmus* spp.*, Nannochlorpsis* spp., *Phaeodactylum tricornutum*, etc. have been used as a renewable source of food, nutraceuticals, animal feed, agrochemicals (Cordeiro et al., 2022). Although the use of seaweed extracts and cyanobacteria in agriculture has been traditionally practiced, the newer developments such as the *omics* approach in microalgae biotechnology and biorefinery approaches in algal biomass utilization have reinforced the applications of microalgae and cyanobacteria in agriculture (Behera et al., 2021).

Microalgal metabolites have been reported to improve soil fertility, impart resistance to plants against abiotic stress, stimulate defense response against pathogens and infection, and improve nutrient uptake from soil such as phosphorus (P), potassium (K), N, and minerals (Berthon et al., 2021; Gonçalves, 2021). Several reports on the use of microalgae for the improvement of crop quality and productivity in various Agri-Horti crops have been published in recent years (Abinandan et al., 2019; Gonçalves, 2021; Kapoore et al., 2021; Lee and Ryu, 2021). Although the different classes of microalgae metabolites with biostimulant and biofertilizer properties have been identified and compiled earlier (Kapoore et al., 2021); however, their mechanisms of action, and impact on plant physiology have not been clearly understood. Further, the effect of a different mode of biostimulant applications on plants, concerning microalgae has been seldom discussed in earlier reports.

A Large quantity of microalgae biomass is the foremost requirement for agriculture applications, especially as plant growth promoters and fertilizers. Despite enormous research on the microalgae biomass production, sustainably achieving high biomass productivity is still far from reality (Calijuri et al., 2022). Commercial production of microalgae throughout the year have been possible only in few tropical and sub-tropical regions offering high light and conducive temperature without affecting conventional agriculture productivity (Correa et al., 2019; Casanova et al., 2022; Rasheed et al., 2022). Use of artificial lighting and controlled photobioreactors (PBRs) for continuous biomass production have been demonstrated (Peter et al., 2022). However, the process is economically unviable owing to the high capital and energy inputs reducing sustainability. Apart from the light requirements, some of the other critical challenges involved in microalgae biomass production are high water footprint, high nutrient costs, and energy-intensive downstream processing (Maiolo et al., 2020; Ighalo et al., 2022). These challenges offset the benefits imparted by the microalgae biomass for agriculture applications necessitating sustainable microalgae bioprocesses. Integration of wastewater bioremediation and flue gas utilization for microalgae cultivation and deployment of biorefinery strategies for biomass utilization have been identified to be sustainable choices (Carraro et al., 2022; Zafar et al., 2022). However, a detailed review of the feasibility of the above processes and the risks involved are not available.

Thus in the present review, we summarize the different types of plant growth-promoting activity viz., biofertilizers, biostimulants, and biopesticides, exhibited by microalgae. In addition, we describe the mechanism of biostimulant action of various microalgae metabolites along with their effects on plants under different modes of application namely seed treatments, foliar spray and soil drenching. Although few reviews and status papers on this topic are available, they seldom discuss the functionalities of various class of biostimulants present in microalgae (Chiaiese et al., 2018; Arahou et al., 2022; González-Pérez et al., 2022). Another important highlight of this review is the description of mechanisms involved in the abiotic and biotic stress tolerances imparted by microalgae on crops along with case studies; which have not been reviewed critically in earlier reports such as (Kapoore et al., 2021; González-Pérez et al., 2022). Further, we address the various challenges involved in the microalgae bioprocesses and describe different strategies such as integration of bioremediation with biomass production and biorefinery approaches to improve the sustainability quotient. We emphasize the need for a closed-loop circular economy model for sustainable agriculture with the case study of integrating microalgae cultivation with modern agriculture technologies such as hydroponics. The main aim of this review is to popularize the commercial use of microalgae-based plant growth additives and highlight the various strategies to combat the challenges involved thereof.

## 2 Growth-promoting properties of microalgae

### 2.1 Microalgae as biofertilizers

Microalgae impart growth-promoting properties in three different modes, namely as biofertilizers, biostimulants, and biopesticides. These properties could be attributed to the presence of a variety of biomolecules such as soluble AAs, phenolic compounds, phytohormone-like compounds, terpenoids, and polysaccharides (Lee and Ryu, 2021). The most common mode of utilization of microalgae biomass is biofertilizers. Biofertilizers are live microorganisms or compounds derived from microbes that enhance or augment plant nutrition by mobilizing or enhancing the nutrient availability in soils by colonizing the rhizosphere, rhizoplane, or root interiors (Mitter et al., 2021). Based on the characteristic functions, biofertilizers can be mainly categorized into a) N-fixing fertilizers b) potassium solubilizing fertilizers c) potassium mobilizing fertilizers, d) phosphate mobilizing and solubilizing fertilizers (Win et al., 2018; Gonçalves, 2021).

Microalgae and cyanobacteria along with fungi and bacteria constitute the uppermost layers of soil collectively called biological soil crust (BSC) which plays a critical role in enhancing soil fertility and crop productivity (Abinandan et al., 2019). Several reports have been published on the presence of microalgae and cyanobacteria in the formation of BSC, especially in a variety of soil types ranging from clay loams, desert soils, semi-arid, silt loam to sandstone and granite (Acea et al., 2003; Malam Issa et al., 2007; Nisha et al., 2007; Wang et al., 2009; Lichner et al., 2013; Manjunath et al., 2016; Renuka et al., 2016; Dineshkumar et al., 2018). These soils are characterized by poor organic (carbon (C) and N and micronutrient content owing to higher surface temperature, Ultra-voilet (UV) irradiation, and elevated carbon dioxide (CO_2_) levels. Despite such harsh environmental conditions and lack of moisture on the soil surface, microalgae and cyanobacteria initiate BSC formation and survive these harsh conditions through adaptive mechanisms such as the formation of heterocysts, secretion of hydrophobic AAs, and extracellular polysaccharides (EPS) and specialty molecules known as phytochelatins that prevent desiccation of intracellular contents, protection from UV-B radiations and degradation of nucleic acids (Garcia-Pichel et al., 2001; Bhargava et al., 2005). A survey of various agroecosystems and soil microbial communities revealed a significant presence of cyanobacteria groups such as Oscillatoriales, Nostocales, Chroococcales, Synechococcales, Chroococcidiopsidales, Pleurocapsales, Microcoleaceae, Chlorellales (Trivedi et al., 2016). Experimental inoculation of microalgae/cyanobacteria in different soil types improved soil nutrients (organic C, N, P, and other minerals) concentration, soil stability, soil moisture content, and water penetration to soil (Acea et al., 2003; Malam Issa et al., 2007; Nisha et al., 2007; Wang et al., 2009; Lichner et al., 2013).

#### 2.1.1 N fixation by cyanobacteria and microalgae

The foremost function exhibited by microalgae, specifically cyanobacteria, as biofertilizer is atmospheric N fixation and enhancement of the soil N content. The most abundant source of N (N_2_) in the earth is atmospheric N, however, they are inert and require high energy for its reduction to ammonia for further uptake by plants. Atmospheric N_2_ enters the biological N cycle in three main ways, viz., through biological fixation (prokaryotic conversion of N_2_ to ammonia); by atmospheric fixation (lightning and photochemical conversion of N_2_ to nitrate); and by the Haber–Bosch industrial process where ammonia is produced from N_2_ (Kraiser et al., 2011). N supplementation in intensive agricultural practice is through the application of N-rich fertilizers such as urea and ammonium sulfate to the soil. However, the major drawback is that only 50% of the N is taken up by the plant while the remaining is lost to the environment due to ammonia volatilization, nitrification, leaching, and surface runoff. This deficit of soil N content can be corrected by the fixation of abundant atmospheric N (Bouwman et al., 2009).

Cyanobacteria have special mechanisms to fix inert atmospheric N. They are diazotrophs and utilize nitrogenase enzyme complex to convert atmospheric N to ammonia at the expense of 16 ATP molecules, a highly energy-intensive process under anoxic (oxygen-free) conditions (Stal, 2015). High light and oxygen exposure inhibits N fixation in cyanobacteria, and they have adopted several strategies for simultaneous photosynthetic and N fixation processes (Gallon, 1992). Among these, the spatial separation of photosynthesis and anaerobic N fixation process between vegetative cells and heterocysts in heterocystous cyanobacteria is one such mechanism (Maldener and Muro-Pastor, 2010). The second mechanism is by temporal separation of photosynthesis and N_2_ fixation process under light and dark regimes in non-heterocystous cyanobacteria exemplified by *Lyngbya* spp., and *Cyanothece* spp., (Misra, 1999). The third mechanism is by the combination of spatial and temporal separation exemplified by *Gleothece* spp., (Compaoré and Stal, 2010 *)* and lastly by genome reduction and loss of oxygenic photosynthesis as observed in *Trichodesmium* spp., (Bergman et al., 2013).

Among the various types of N_2_ fixing mechanisms, the most predominant and commonly observed in terrestrial ecosystems is heterocyst based N_2_ fixation. This process happens through a symbiotic relationship with the host plant where cyanobacterial species colonize the leaf and roots of the host plant (Krings et al., 2009). Cyanobacteria enter the leaf tissue through the stomata and colonize the intercellular spaces by forming a cyanobacterial loop while in the case of roots, they form loose colonies on the root hair and tight colonies on the root surfaces (Lee and Ryu, 2021). Some examples include the colonization of roots of wheat and cotton by *Anabaena* spp., and *Tolypothrix* spp., and rice by *Nostoc* spp., (Babu et al., 2015). The colonization of roots and further process by cyanobacteria is called ‘Gland formation’ and the process has been elucidated and the mechanism is similar to the nodule formation by *Rhizobium* spp. or gall formation by *Agrobacterium tumefaciens*. The process involves cell penetration, intracellular colonization, hormogonium formation, and gland development with host specificity (Santi et al., 2013). Microalgae as a source of N_2_ is applied to the soil as live culture in case of cyanobacteria or as dried biomass or suspension in the case of green algae (Alvarez et al., 2021). The major advantage of the use of microalgae in soil as a source of N is that there is lesser chance of leaching or loss as run offs unlike chemical based N fertilizers since less than 5% of N content in microalgae biomass is in the mineralized form (Mulbry et al., 2005). Further, the issue of NH_3_ volatilization is negligible with dried algae biomass application unlike urea or other manures (de Siqueira Castro et al., 2017).

#### 2.1.2 Phosphorus solubilization

Apart from N, P exists as the second most limiting nutrient for plant growth. P exist in the soil in the form of inorganic phosphates or in complex organic forms making them unavailable to plants, necessitating the use of P-rich fertilizers (Elser, 2012). Despite the enormous use of P fertilizer in agro-production only a portion of the P is available to plants as the significant amount is lost due to erosion and leaching leading to the contamination of groundwater and water eutrophication (Cordell et al., 2009). The safe and effective alternative that can cut down the overuse of P fertilizers in crop production is use of phosphate solubilizing microbes (PSM) and biofertilizers that augment P uptake from soil (Gyaneshwar et al., 2002). Among the PSM, cyanobacteria and microalgae play a critical role in phosphate solubilization to plants. Depending on the soil pH, the P is bound to calcium (Ca) or aluminium in soil. Cyanobacteria solubilize bound P in two ways either by releasing chelators that bind Ca ions or by releasing organic acids that promote solubilization (Alvarez et al., 2021). Experimental observations revealed that cyanobacterial species such as *Anabaena variabilis* and W*estelliopsis* spp., secrete pthalic acid for P solubilization from phosphate rock and tricalcium phosphate (Yandigeri et al., 2011). In addition to solubilization, cyanobacteria and microalgae mineralize P from organic P sources. The most commonly occurring organic P sources are phytates and phosphoesters and microalgae produce P hydrolyzing enzymes such as alkaline phosphatases, phosphodiesteratses, 5’nucleotidases and phytases that release bound P from organic molecules (Markou et al., 2014).

Further, microalgae and cyanobacteria demonstrate a luxury uptake mechanism where they accumulate intracellular P reserves as polyphosphate granules. This P reserve is utilized by the algae when the P levels in the surrounding medium is depleted (Powell et al., 2009). This luxury uptake mechanism is further supported by additional physiological processes such as membrane lipid remodeling to reallocate P based on its availability from surrounding medium. This is achieved through modulation of lipid composition, where P starvation induces remodeling of membrane lipid moieties such as phosphatidylethanolamine, phosphatidylcholine, and phosphatidylglycerol (phospholipids) to non-P containing glycolipids or betaine class of lipids such as sulfoquinovosyldiacylglycerol or diacylglyceroltrimethylhomoserine leading to P reallocation (Çakirsoy et al., 2022). Such physiological mechanisms offer microalgae to accommodate additional P from surrounding medium or reallocate P during P deficiency by way of polar lipid remodeling as observed in few fast growing and high P uptake microalgae species such as *Nanochloropsis oceania, Nannochloropsis gaditana, Tetraselmis suecia* (Cañavate et al., 2017).This phenomenon of luxury uptake of P by microalgae could be utilized for delivering soluble P to plants.

#### 2.1.3 Enhancing micronutrient bioavailability

In addition to aforesaid macronutrients, minerals such as iron (Fe) play a crucial role in the growth of plants. The typical Fe requirement of plants is in the order of 10^-6^ moles (M). Although Fe is abundant in soil, their bioavailability to plants is negligible owing to physico-chemical properties of soil. Under aerated conditions and soil pH > 7.00, the inorganic Fe becomes poorly soluble and bioavailable Fe concentration is in the range of 10^-10^ M leading to Fe scarcity (Colombo et al., 2014). To counter this problem, soil and rhizosphere bacteria release low molecular weight, organic molecules called siderophores. Siderophores are nitrogenous compounds with strong affinity for Fe^3+^ ions and contribute to the solubilization and mobilization of Fe into plants (Chakraborty et al., 2019). Similar to bacteria, microalgae and cyanobacteria secrete siderophores (Årstøl and Hohmann-Marriott, 2019).

Siderophores form a strong hexadentate, octahedral complex with Fe^3+^ ion. Based on the primary oxygen donating ligands, siderophores are primarily classified into hydroxamates, catecholates, and carboxylates (Hider and Kong, 2010). The predominant class of siderophores observed in cyanobacteria are hydroxamates. The two hydroxamate siderophores whose structure has been determined are schizokinen and synechobactin in *Anabaena* spp., and *Synechococcus* spp., respectively (Simpson and Neilands, 1976). The structure of the cyanobacterial siderophore is similar to the bacterial siderophore rhizobactin and aerobactin (Årstøl and Hohmann-Marriott, 2019). Additionally, a catecholates group of siderophores named anachelin has been detected in *Anabaena cylindrica* which chelates Fe^3+^ ions through a catechol moiety (Beiderbeck et al., 2000). The presence of siderophore not only helps in Fe binding and mobilization but also in preventing heavy metal toxicity to the algae. In *Anabaena* spp., PCC 7120, under high copper conditions (copper toxicity) chelation of Cu ions by siderophores was observed. Cellular recycling of siderophores results in exclusion of Cu ions reducing the toxicity of Cu ions to cyanobacterial cells (Clarke et al., 1987). This mechanism can be exploited in heavy metal sequestration in contaminated soils. Few observations on the ability of cyanobacterial hydroxamates to sequester heavy metals have been reported such as such as uranium sequestration by *Synechococcus elongates* BDU 130911 and cadmium chelation by *Anabaena oryzae* under Fe replete conditions (Rashmi et al., 2013; Singh et al., 2016). Thus cyanobacteria have wide applications as natural biofertilizers in replenishing of soil nutrients and their mobilization to plants.

### 2.2 Microalgae as biostimulants

Biostimulants are compounds other than fertilizers that enhance the crop productivity by acting directly on the plants regardless of its nutrient content (Du Jardin, 2015). These are group of organic compounds that enhance the crop productivity by increasing the nutrient uptake in plants, imparting resistance to various biotic and abiotic stresses, improve soil water use efficiency, reinforcement of root system, and maintenance of physiological processes such as respiration, photosynthetic activity, Fe uptake and nucleic acid synthesis (Du Jardin, 2015; Lee and Ryu, 2021; Kumar et al., 2022d). Microalgae have been identified with several biostimulatory compounds such as phenolics, phytohormones mimicking compounds, terpenoids, polysaccharides and AAs (Ronga et al., 2019; Gonçalves, 2021). Extracts and metabolites obtained from microalgae species such as *Chlorella* spp., *Spirulina platensis*, *Acutodesmus* spp., *Scenedesmus* spp., *Dunaliella* spp., *Calothrix elenkini* etc. are commonly used as biostimulants (Ronga et al., 2019; Colla and Rouphael, 2020).

#### 2.2.1 Phytohormones mimicking compounds from microalgae

Phytohormones are a low molecular weight, structurally unrelated signaling molecules that occur naturally in plants and provide stimulatory effects at very low concentrations in the various plant development processes such as root and shoot formation, tissue differentiation, fertilization and plant senescence, defense and tolerance to various biotic and abiotic stresses (Gray, 2004; Santner et al., 2009; Fahad et al., 2015). Based on their functions, the phytohormones are classified into auxins, cytokinins, gibberellic, abscisic acid (ABA) and ethylene.

##### 2.2.1.1 Auxins

Auxins are tryptophan derived plant hormones involved in regulation of key physiological processes such as cell division and elongation, vascular tissue differentiation, tropism apical dominance and stress response (Wang et al., 2001; Eyidogan et al., 2012). They are the first identified class of phytohormones, consisting compounds like indole-3-acetic acid (IAA), 4-chloroindole-3-acetic acid, indole-3-butyric acid (IBA), indol-3-acetamide (IAM), and 2-phenylacetic acid (Górka et al., 2015). Microalgae such as *Chlorella* spp., *Coenochloris* spp., *Acutodesmus* spp., and *Scenedesmus* spp., *Chlorococcum* spp., were found to contain auxins in the concentration ranging between 0.18 to 99.83 nmol g^-1^ dry weight (DW) biomass consisting two major compounds viz., IAA and IAM. However, IAA was the predominant auxin detected in almost 24 microalgae species (Stirk et al., 2013b; Kapoore et al., 2021). The direct role of IAA derived from microalgae extracts in root formation and elongation was observed with *Petunia x hybrida* plants. Foliar spray (FS) of extracts obtained from *Scenedesmus* spp., on *Petunia* plants resulted in increased dry root weight of plants. Analysis of the extracts indicated the presence of IAA at a concentration of 5965 ng g^-1^ (Plaza et al., 2018). The auxins, specifically IAA, have been attributed with the role as signaling molecule during plant cyanobacteria interactions especially for root colonization (Lee and Ryu, 2021). The IAA produced in cyanobacterial species such as *Nostoc* spp., *Synechocystis* spp., *Leptolyngbya* spp., have been found to improve the growth in rice and wheat plants and were found in highest concentrations during colonization of plant roots (Hussain et al., 2013; Hussain et al., 2015). In addition to this, auxins (IAA) and soluble AAs secreted by cyanobacteria have been identified to enhance soil microbial content and micro biome quality (Karthikeyan et al., 2009; Lee and Ryu, 2021).

##### 2.2.1.2 Cytokinins

Cytokinins are N6-substituted adenine derivatives containing either aromatic or isoprenoid side chains (Santner et al., 2009). They play a significant role in plant developmental processes such as shoot differentiation, cell division, nutrient mobilization, photo-morphogenic development, chloroplast biogenesis, apical dominance and vascular differentiation (Fahad et al., 2015). The most predominant cytokinins observed in microalgae are zeatin, zeatin riboside, kinetin, isopentenyladenosine (Górka et al., 2015). The cytokinins content ranged between 0.29 nmol g^-1^ DW and 21.40 nmol g^-1^ DW with maximum concentration observed in *Stigeoclonium nanum. cis*-Zeatin and isopentenyl adenine were the predominant cytokinins while trans-zeatin and dihydrozeatin were found in low concentrations in addition to free bases, ribosides (Stirk et al., 2013b). Apart from physiological and developmental functions, microalgae derived cytokinins have been reported to impart abiotic stress tolerance in host plants. For example, use of cytokinin containing extracts of *Nannochloropsis* spp., alleviated water and N stress in tomato plants (Oancea et al., 2013; Lu et al., 2014). The putative mechanism behind the stress tolerance in plants induced by microalgae cytokinins could be attributed to the free radical scavenging properties of cytokinins (Fahad et al., 2015).

##### 2.2.1.3 Gibberellic acids

Gibberellic acids (GA) are specific class of phytohormones with main functions in abiotic stress tolerance in plants (Fahad et al., 2015). GAs modulate photosynthetic efficiency of plants and promote the redistribution of photosynthesis, thus balancing source – sink relationship during abiotic stress (Iqbal et al., 2011). About 19 different types of GAs have been identified in microalgae with main functions of stem elongation, initiation of seed germination *via* enzyme activation (alpha-amylase), initiation of flowering, floral organ development and influencing protein biosynthesis (Kapoore et al., 2021). The total concentration of GAs in microalgae range between 3 pg mg^-1^ biomass for GA7 in *Gyoerffyana humicola* to 3452.9 pg mg^-1^ for GA15 in *Scotiellopsis terrestris* (Stirk et al., 2013a). It has been observed that extracts containing GA3 derived from *Chlorella vulgaris* reduced the adverse effects caused by heavy metal stress and impart defense against lead and Cadmium (Han et al., 2018).

##### 2.2.1.4 Ethylene

Ethylene is a gaseous hormone that regulates developmental processes such as senescence, fruit ripening, cell division and elongation, and tolerance to biotic and biotic stresses (Han et al., 2018). Microalgae species belonging to the genus *Chlamydomonas*, *Chlorella*, *Scenedesmus* and cyanobacteria such as *Synechococcus* spp., *Anabaena* spp., *Nostoc* spp., *Calothrix* spp., *Scytonema* spp., and *Cylindrospermum* spp., have been reported to synthesize ethylene (Lu and Xu, 2015). Plaza et al. (2018) reported ethylene content of 341 ng g^-1^ DW and 546 ng g^-1^ DW in *Scenedemsus* spp., and *Spirulina platensis* respectively.

##### 2.2.1.5 Abscisic acid

Abscisic acid is a C_15_ sesquiterpenoid hormone which play an important role in the adaptive responses of plants to various biotic and abiotic stresses. They function by modulating stomatal closure, biosynthesis of proteins and compatible solutes/osmolytes, and maintenance of water status in plants enabling tolerance in plants to stressors (Eyidogan et al., 2012). In general, they are general inhibitors of growth and metabolic functions and functions in conjunction with other phytohormones or signaling molecules such as auxins, cytokinins, ethylene and brassinosteroids (Fahad et al., 2015).

#### 2.2.2 Other hormone like signaling molecules

In addition to phytohormones, microalgae and cyanobacteria accumulate low molecular weight signaling molecules such as brassinosteroids, polyamines and jasmonic and salicylic acids (Kapoore et al., 2021; Lee and Ryu, 2021). Brassinosteroids are steroidal plant hormones that either exist freely or are conjugated to sugars or fatty acids with primary function in seed germination, vascular differentiation, leaf bending and pollen tube elongation (Fahad et al., 2015). They have been associated with the stress response mechanisms of plants, especially in the tolerance to salt stress and enhance the enzymatic and non-enzymatic defence response in stressed plants (Sharma et al., 2013). Microalgae have been identified with two types of brassinosteroids, viz., brassinolide and catasterone. The brassinosteroid contents ranged from 117.3 pg g^-1^ DW in *Raphidocelis subcapitata* MACC 317 to 977.8 pg g^-1^ DW in *Klebsormidium flaccidum* MACC 692 (Stirk et al., 2013b).

Jasmonic (JA) and Salicylic acids (SA) are ubiquitous messenger/signaling molecules in plant defence systems with a significant role in biotic stress response. They play a critical role in seed germination, glycolysis, flowering, upregulation of antioxidant genes, ion uptake and transport, photosynthetic rate, stomatal conductance, transpiration, thermo-tolerance, senescence and nodulation (Fahad et al., 2015). The SA and JA signaling pathway are interconnected with phytohormones signaling and they act antagonistically to auxins, cytokinins and GA responses while acting synergistically with ethylene and ABA (Kapoore et al., 2021). JA levels increase in response to various inductive signals such as mechanical wound, herbivory and abiotic stresses while SA levels increase with infection of the host plants by a broad range of pathogens (Santner et al., 2009). Jasmonic and salicylic acids have been detected in most of the microalgae species, and in significant quantities in *Scenedesmus* spp., 75.13 ng g^-1^ and 156714 ng g^-1^ for JA and SA respectively (Plaza et al., 2018; Kapoore et al., 2021).

Polyamines are low molecular weight poly-cations that play a prominent role in plant growth and development processes and stress responses (Kusano et al., 2008; Chen et al., 2019). The most common forms of polyamines are putrescine, spermidine and spermine (Mustafavi et al., 2018). Among the various microalgae species, *Spirulina platensis* contained significant quantities of polyamines *viz*., 0.76 μg of putrescine, 3.31 μg of spermine and 0.67 μg of spermidine per gram dry biomass (Tarakhovskaya et al., 2007). The polyamines of *Spirulina* have been reported to enhance the growth of lettuce seedlings, exhibiting a biostimulant behavior (Mógor et al., 2018a).

#### 2.2.3 Microalgal polysaccharides as bio stimulatory compounds

Polysaccharides are complex macromolecular polymers of neutral sugars with diverse compositions with varied degree of polymerization, chemical substitution and biological activity (Chanda et al., 2019). Polysaccharides stimulate plant growth and metabolism by modulating physiological and biochemical processes. Some of the stimulatory activities exhibited by polysaccharides are enhancement of root growth, nutrient availability and mobilization through chelation of minerals, and enhancement of photosynthesis through increased synthesis of Rubisco and tolerance to biotic and abiotic stress and act as signaling molecules (Moreira et al., 2022).

The typical mechanism by which microalgal polysaccharides exhibit stimulatory properties is through microbial associated molecular patterns dependent signaling pathways (Chanda et al., 2019). The mechanism has been elucidated in seaweed polysaccharide extracts (Mukherjee and Patel, 2020) which can be well extrapolated to microalgal polysaccharides. Briefly, the complex polysaccharides are hydrolyzed by soil enzymes such as beta glucanase, chitinase secreted by microorganisms and these neutral sugars from polysaccharides could be recognized by the receptors on plant membranes as microbial derived compounds that induce signaling cascades by (i) activation of Ca2+ influx, (ii) stimulation of octadecanoid and phenylpropanoid pathways leading through the enzymes lipoxygenase (LOX) and phenylalanine ammonia lyase (PAL), (iii) activation of SA and JA signaling pathway, (iv) reactive oxygen species (ROS) scavenging enzymes such as catalase (CAT), peroxidase (POD) and superoxide dismutase (SOD) and synthesis of phenolic and secondary metabolites that act as defense molecules (Chanda et al., 2019; Farid et al., 2019). Among the various microalgae species, cyanobacteria such as *Spirulina platensis, Nostoc* spp.*, Phormidium* spp.*, Calothrix* spp.*, Plectonema* spp., are known to produce polysaccharides that are secreted to the surrounding medium and termed as exopolysaccharides (EPS) (Parwani et al., 2021). Similarly, eukaryotic microalgae such as *Chlorella vulgaris, Chlorella stigmatophora, Porphyridium cruentum, Tetraselmis* spp.*, Dunaliella salina* produce polysaccharides that have biostimulatory potential (Chanda et al., 2019).

The EPS secreted by cyanobacteria have various benefits in terms of enhancing crop productivity. The foremost of them is the bioadhesive property of EPS that results in the formation of microbial mats and biofilms promoting BSC formation (Rossi and De Philippis, 2015; Abinandan et al., 2019). Further, cyanobacterial EPS have soil conditioning properties where they promote the formation of micro-aggregates of soil leading to moisture retention, nutrient accumulation and proliferation of soil microflora (Rossi and De Philippis, 2015). EPS-secreting cyanobacterial biofilms are concentrated source of nutrients and offer benefits such as mineralization of complex soil nutrients, C and N fixation in soil, protection against drought and desiccation and heavy metal sequestration (Garlapati et al., 2019; Parwani et al., 2021; Moreira et al., 2022).

Field applications of cyanobacteria and microalgae polysaccharides have shown beneficial effects in host plants. Foliar application of polysaccharide-rich extracts from *Spirulina platensis* resulted in enhanced growth of tomato and pepper plants (Elarroussia et al., 2016). Similarly, polysaccharide extracts of *Chlorella vulgaris, Chlorella sorokiniana*, *Chlamydomonas reinhardtii*, *Dunaliella salina* had shown bio stimulatory properties when injected into tomato plant seedlings (Farid et al., 2019). The group observed that polysaccharide extracts of above mentioned green microalgae enhanced the activity of defence enzymes such as LOX, PAL, and ROS scavenging antioxidant enzymes such as CAT, POD and ascorbate peroxidase (APX). In addition, they enhanced very long chain fatty acids in the leaves of tomato plant which constitute the cuticular wax composition indicating activation of plant defence mechanisms against external stressful stimuli. In another study, El Arroussi et al. (2018) reported enhanced stress tolerance to salinity in tomato plants when applied with EPS obtained from *Dunaliella salina*. The literature amply demonstrates that polysaccharides from microalgae and cyanobacteria have plant growth promotion and biostimulatory properties that can be exploited for enhanced crop productivity.

#### 2.2.4 Other microalgae metabolites in crop productivity enhancement

Similar to polysaccharides, protein hydrolysates, peptides and free AAs obtained from microalgae have been reported to enhance crop productivity (Kapoore et al., 2021). The primary function of these hydrolysates and AAs are nutrient mobilization into plants through complexation and chelation of essential minerals (Du Jardin, 2015). Additionally, these AAs play a critical role in abiotic stress mitigation by acting as osmoprotectants and antioxidants such as glycine, betaine and proline against environmental stress such as heavy metals and salinity (Bulgari et al., 2015). Further, application of AAs and protein hydrolysates enhanced the plant growth promoting bacteria by acting as a source of reduced N to the microflora thus promoting the soil microbiome (Colla et al., 2017; Lee and Ryu, 2021). The protein content of microalgae and cyanobacteria range up to 63% with AAs contents constituting between 40% and 48% of total proteins (Hempel et al., 2012; Kumar et al., 2022c). High amounts of certain AAs like arginine and tryptophan in species such as *Spirulina platensis* make them an attractive option for biostimulant application as they act as precursors for polyamines and auxins respectively (Bulgari et al., 2019). Foliar application of protein rich extracts of *Spirulina platensis* on red beet increased the hypocotyl growth, chlorophyll and nutrient composition (Mógor et al., 2018b). while in case of *Petunia x hybrida* the number of flowers, flower fresh and DW was enhanced (Plaza et al., 2018). In another study, application of AA rich extracts of green microalgae enhanced the solids content, total organic and capsaicinoids content in three varieties of hot pepper (*Capsicum* spp.). The study suggested variety specific outcome with respect to biostimulatory treatments (Zamljen et al., 2021).

Antioxidants and micronutrients are another important group of nutrients offered by microalgae and cyanobacteria towards enhancing crop productivity. Some of the important class of micronutrients are vitamins, specifically ascorbic acid that impart tolerance to both biotic and abiotic stresses (Kapoore et al., 2021). Among the antioxidants like phenols, terpenoids and carotenoids, the foremost group is carotenoids that play a central role in photosynthesis and photoprotection. In addition, carotenoids contribute to the pigmentation of seeds, fruits, and flowers and act as precursors for plant signaling hormones such as ABA and strigolactone (Sun et al., 2022). The prominent group of antioxidants found in microalgae are carotenoids such as alpha and beta carotene in *Dunaliella salina*, *Chlorella vulgaris*, fucoxanthin in *Phaeodactylum tricornutum* and *Isochrysis cabana*. The carotenoid composition of microalgae and cyanobacteria have been well reviewed by several authors previously (Vidyashankar et al., 2017; Cezare-Gomes et al., 2019). Although the role of carotenoids in plant growth and development is characterized, the mechanisms of carotenoid conversion and uptake from microalgae biomass is not elucidated requiring further detailed studies.

#### 2.2.5 Microalgae phenolics as biostimulants

Phenolics are another important group of metabolites that play a critical role in stress signaling and defence responses to infection and injury (Mandal et al., 2010). Phenolic compounds have been attributed to various defence mechanisms observed in plants in response to abiotic stresses. In addition, phenolics increase nutrient absorption by chelating ions and mobilize the uptake of nutrients such as Ca, zinc, and Fe leading to enhanced porosity of soils (Perera and Tirimanne, 2021). Phenolic acids and flavonoids act as sunscreens protecting plants from UV-B radiation, specifically kaempferol and derivatives (Tamrat Alemu and Roro, 2020). Further, they are involved in drought, salinity and heavy metal stress tolerance with a primary role of scavenging ROS generated during oxidative stress. These abiotic stresses generate hydrogen peroxides, hydroxyl and superoxide ions that act as free radicals (Dehghanian et al., 2022). In addition to abiotic stress, phenolic acids are generated as a defence response to insects, phytopathogens and herbivory (Dehghanian et al., 2022). Phenolic acids and derivatives such as hydroxycinnamate conjugates and hydroxycoumarins are produced during infections from phytopathogens (Kumar et al., 2020). Microalgal polyphenols and flavonoids are less explored compared to the other metabolites in them. The total polyphenol content of microalgae ranged between 0.16 mg gallic acid equivalent (GAE) g^-1^ in *Neochloris* spp., to 60 mg GAE g^-1^ in *Nostoc*. spp., while flavonoids ranged between 0.84 mg Quercetin equivalent (QE) g^-1^ in *Phaeodactylum* to 4.03 mg QE g^-1^ in *Desmodesmus* spp., (Del Mondo et al., 2021). Gallic, ferulic, caffeic, chlorogenic, synapic and coumaric acids and hydroxybenzoates are commonly found phenolic while in the case of flavonoids, rutin, quercetin, kaempferol are predominantly observed among different microalgae species (Del Mondo et al., 2021).

#### 2.2.6 C-phycocyanin as biostimulant

**Figure 1:**
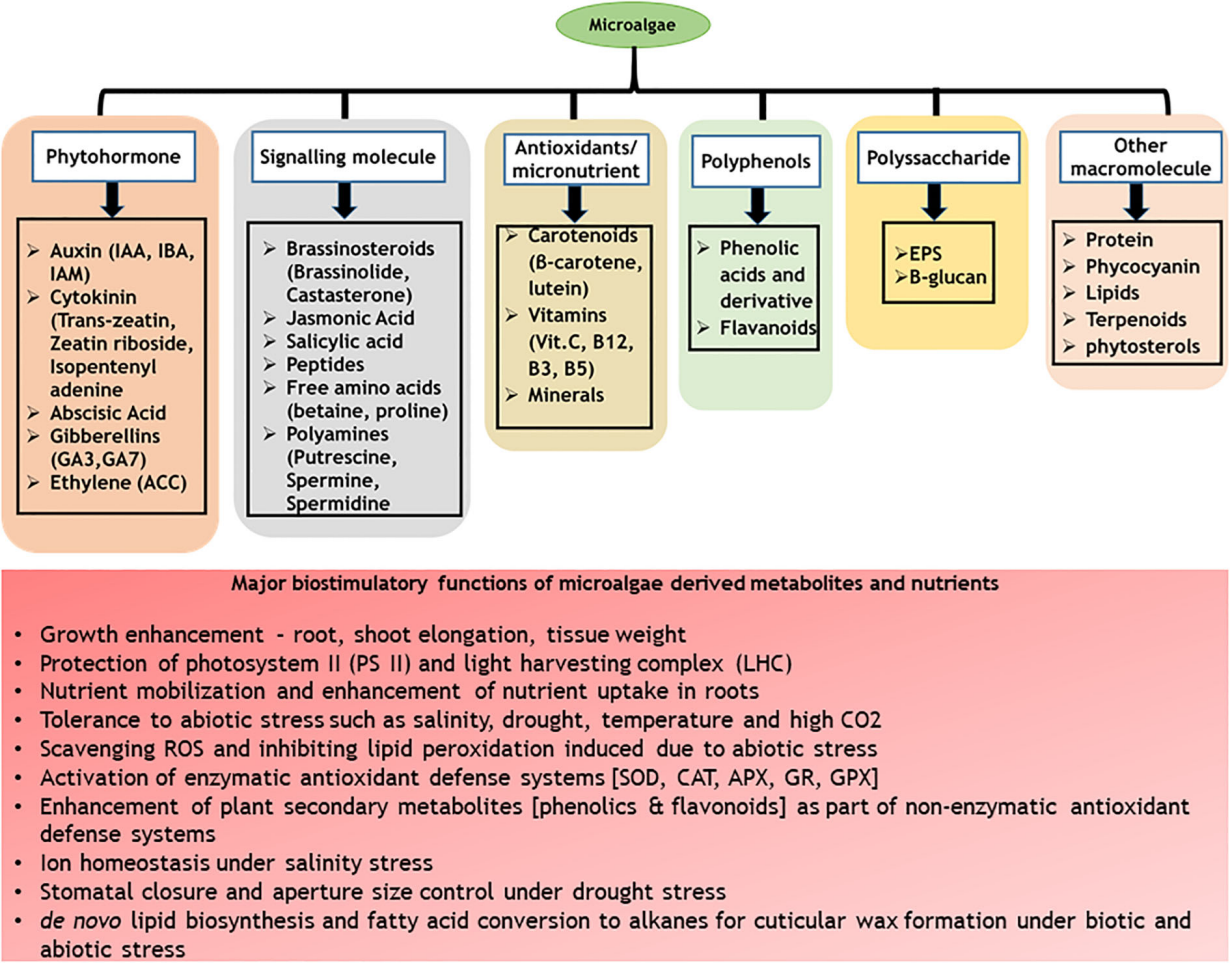
A summary of various bio stimulatory compounds present in microalgae and cyanobacteria.

C-phycocyanin (CPC) is a water-soluble phycobiliprotein (protein-pigment complex) majorly distributed in cyanobacteria with light-harvesting functions. Cyanobacteria such as *Spirulina platensis*, *Phormidium* spp., *Nostoc* spp., *Anabaena fertilissima* PUPCCC 410.5, *Synechocystis* spp., and *Galdieria sulphuraria* are known to contain significant content of CPC (Kaur et al., 2019; Avci and Haznedaroglu, 2022). Commercially, CPC is produced from *Spirulina platensis* towards food (as a natural blue colourant) and therapeutic applications (as an antioxidant) (Patel et al., 2022). Apart from the nutraceutical applications, CPC has been recently identified with plant biostimulant properties (Varia et al., 2022). Treatment of tomato seeds with CPC enhanced the germination index, shoot and radicle length along with the modulation of chemical composition, specifically secondary metabolites such as phenolics and flavonoids (Metwally et al., 2022). Application of CPC in vertical hydroponic systems promoted early maturity in lettuce along with enhanced biomass productivity, leaf diameter and total flavonoids (quercetin and luteolin) content (Varia et al., 2022). In addition to these, CPC modulated microbial diversity and abundance in the hydroponic growth medium promoting actinobacteria and firmicutes suggesting possible plant prebiotic properties of CPC by its ability to stabilize plant growth promoting bacteria and enhancing plant growth (Varia et al., 2022). The major advantage of use of CPC as biostimulants is that the molecule is well characterized along with its downstream processes such as extraction and purification well standardized and commercialized (Arahou et al., 2022). Further, CPC is water soluble in nature allowing its scalability as bio stimulants. The major bottleneck in the use of CPC in open conditions is its light-sensitive nature and poor half-life at high light intensities (Adjali et al., 2021). However, under controlled atmospheric growth conditions such as hydroponics and other vertical farming systems, CPC can be well exploited as biostimulants. A summary of various bio stimulatory compounds present in microalgae and cyanobacteria and their functions are presented in **Figure 1**.

#### 2.2.7 Micronutrients from microalgae biomass

Microalgae are rich source of micronutrients like K, Ca, P and trace elements such as, iron, zinc, copper and manganese. The total P content of the biomass ranged between 0.73% to 1.46% w/w with highest content observed in marine microalgae *Tetraselmis chuii* and freshwater oleaginous microalga *Botryococcus braunii* (1.45 - 1.46% w/w) (Tibbetts et al., 2015). The Ca content ranged between 0.1% to 2.9% w/w with highest content observed in *Tetraselmis chuii* (2.99% w/w) and *Phaeodactylum tricornutum* (2.91% w/w) followed by *Porphyridium cruentum* (2.06% w/w) (Tibbetts et al., 2015; Di Lena et al., 2020). The K content was highest in *Phaeodactylum tricornutum* (2.4% w/w) and other microalgae species containing K ranging from 0.7% w/w to 1.8% w/w (Tibbetts et al., 2015; Di Lena et al., 2020). Among the most commonly cultivated microalgae viz., *Spirulina platensis* and *Chlorella vulgaris*, the P content was twofold higher in *Chlorella* in comparison with *Spirulina* while in case of K, the trend was vice *versa* with *Spirulina* containing thirty-fold higher K (1.3% w/w - 1.5% w/w) in comparison to *Chlorella* (0.049% w/w) and Ca content ranging between 0.59% and 0.89% w/w (Tokuşoglu and Üunal, 2003). Among the trace elements, Fe was highest in *Porphyridium aerugineum* (1110 mg 100g^-1^) followed by *Botryococcus braunii* (620.31 mg 100g^-1^) (Tibbetts et al., 2015). Among freshwater microalgae, *Chlorella* spp., (259.1 mg 100g^-1^) contained two-fold higher Fe content compared to *Spirulina* (103.6 mg 100g^-1^) (Tokuşoglu and Üunal, 2003). Other trace elements such as Zn, Mn and Cu ranged between 1.5 mg to 13 mg 100g^-1^ (Tokuşoglu and Üunal, 2003; Tibbetts et al., 2015; Di Lena et al., 2020). As discussed in previous section, microalgae accumulate P and Fe using special mechanisms and store as polyphosphate granules and Fe reservoirs respectively (Powell et al., 2009). The mechanism of luxury uptake of minerals observed in microalgae could be exploited for agriculture applications. Microalgae passively bio-absorb nutrients especially, trace metals like Zn, molybdenum (Mo), selenium (Se), Cu, etc. The surface composition of microalgae cell contains charged polysaccharide molecules with charged moieties like carboxyl, sulfonic acids, hydroxyl ions that in turn bind to metal ions and form complexes. Microalgae absorb these metals/charged ions through ion exchange and complexation mechanisms (Michalak and Chojnacka, 2015).

Apart from minerals, microalgae synthesize vitamins that act as growth promoting factors for plants. Among the various water soluble vitamins evaluated, vitamin C was the most abundant, ranging between 100.2 mg kg^-1^ in *Chlorella stigmatophora* and 191 mg kg^-1^ in *Tetraselmis suecia* compared to other B complex vitamins in marine microalgae biomass (Fabregas and Herrero, 1990). Among the vitamin B complex, nicotinic acid was highest ranging between 77.7 mg kg^-1^ in *Isochrysis galbana* to 89.3 mg kg^-1^ in *Tetraselmis suecia* (Fabregas and Herrero, 1990). In case of freshwater microalgae, riboflavin, niacin, folic acid and cyanocobalamin were predominant vitamins present in microalgae such as *Spirulina platensis*, *Chlorella* spp. (Edelmann et al., 2019). The riboflavin content varied between 21 and 41 μg g^-1^ while niacin ranged from 0.13 to 0.28 mg g^-1^ DW respectively, in the aforesaid microalgae species. The folic acid content was six times higher in *Chlorella* spp., 19.7 μg g^-1^ compared to *Spirulina platensis* 3.5 μg g^-1^ (Edelmann et al., 2019). Watanabe et al. (2013) reported presence of true form of vitamin B_12_ in *Chlorella vulgaris* while *Spirulina platensis* and other cyanobacteria such as *Nostoc commune*, *Nostoc flagelliforme* and *Nostochopsis* spp., contained a corrinoid compound or pseudo form of vitamin B_12_ [adeninyl cobalamin] that are not biologically active in mammals. However, these compounds could be potentially bioactive for plant health and plants may utilize these corrinoid compounds as precursors or signaling molecules. Application of vitamin B complex rich microalgae biomass to the soil enhance the vitamin contents of the plant tissues as plants have known to absorb vitamin B12 and other B complex vitamins through roots as evidenced by use of bio fertilizers in cultivation of soybean, spinach and barley (Mozafar, 1994). Apart from micronutrients, microalgae produce metabolites such as terpenoids, humic substances, betains, and peptide (cyanotoins) that act as messenger/signaling molecules, bio stimulants or as allelochemicals that inhibit growth of weeds and microbes and function as bio pesticides (Kapoore et al., 2021).

## 3 Methods of application of microalgae-based bio fertilizer and biostimulants

Cyanobacteria and microalgae, either in the form of live biomass or dried biomass have been applied as biofertilizers while their cellular extracts and hydrolysates as biostimulants in enhancing plant growth and crop yield. Admixtures of soil with live or dry algae biomass, dipping of seeds with cell extracts of microalgae (seed priming), and root drenching are some of the common methods of biostimulant/biofertilizer application (Lee and Ryu, 2021). The mode of application of biostimulants is dependent upon the particular need of the crops such as nutrient supplementation or for micronutrient enrichment or disease suppression. A further type of crop greatly influences the mode of application as whether they are direct seed sown or nursery grown and transplanted in the field (Renuka et al., 2018).

### 3.1 Seed treatments

Seed treatment with biostimulants generally involves three different processes, namely seed priming, seed coating and seed dipping (Gupta et al., 2022). Seed priming is a pre-sowing treatment where the seeds are hydrated in a controlled manner such that the radicle does not protrude. This technique enhances seed germination rate and enhances root formation in plants (Sharma et al., 2014). In seed coating, the seeds are sprayed or coated with biostimulants to form a uniform layer while in seed dipping or soaking treatments, the seeds are soaked for a definite time (18-24 h) prior to sowing. The major advantage with these methods is that it gives a head start in germination and consequently enhances germination index, seedling vigor, increased shoot and radicle length and reduction in harmful seed micro flora (Rocha et al., 2019).

The use of microalgae extracts and live cell suspension in seed treatments resulted in enhanced growth of plants in a variety of crops such as cereals, vegetables and spices. In a recent study, priming of spinach seeds with whole cell extracts and cell lysates of green microalgae *Chlorococcum* spp., *Micractinium* spp., *Scenedesmus* spp., and *Chlorella* spp., resulted in enhanced seed germination, faster cotyledon emergence and seedlings weight (Rupawalla et al., 2022). Similarly, priming of seeds of vegetable crops such as tomato, lettuce and cucumber with cellular extracts of *Spirulina* spp., *Chlorella* spp., *Chlorella vulgaris, Scendesmus* spp., *Synechocystis* spp., and *Acutodesmus obliquus* resulted in higher germination rate compared to untreated seeds (Garcia-Gonzalez and Sommerfeld, 2016; Bumandalai and Tserennadmid, 2019; Supraja et al., 2020a). In addition to enhancing the germination rate and seedling vigour, hydration of seeds with extracts obtained from *Chlorella vulgaris* and *Scenedesmus quadricauda* resulted in increased root length, root diameter and root surface area and the number of root tips in sugar beet (Puglisi et al., 2022). Similar observations were made with cereal crops namely maize and wheat when extracts and cell-free supernatants of cyanobacteria such as *Anabaena* sp. PCC 7120, *Calothrix* spp., *Hapalosiphon* spp., *Nostoc* spp., and *Westiellopsis* spp were applied to the seeds (Karthikeyan et al., 2009; Grzesik and Romanowska-Duda, 2014). Apart from the application of microalgae extracts, inoculation of live cyanobacteria suspensions like *Anabaena laxa* and *Calothrix elenkinii* with the seeds of spice crops such as pepper, coriander, fennel and cumin promoted germination and enhanced root and shoot formation (Guzmán-Murillo et al., 2013; Kumar et al., 2013). The ability of microalgae cell suspensions and cell-free supernatants in enhancing the growth of seedlings, germination rate and root formation could be attributed to the presence of EPS in the supernatant while that of microalgal extracts could be attributed to the presence of phytohormones such as cytokinins (trans-Zeatin, dihydrozeatin, isopentyladenine and kinetin), gibberellins (GA1, GA3, GA4, GA20 and GA29), auxin (IAA) and ABA (Rupawalla et al., 2022).

### 3.2 Foliar spray

Foliar spray of biostimulants is a commonly employed method for enhancing crop productivity in several crops owing to the faster response of plants to nutrients supplemented compared to other treatments (Arahou et al., 2022). FS of biostimulants enhanced the water use efficiency and stomatal functioning of the plants (Renuka et al., 2018). Although widely used method, the mechanism of uptake of biostimulants and nutrients through FS is still not clearly understood. Four major pathways of entry have been hypothesized namely through cuticle cracks, stomata, aqueous and ectodesmatal pores (Ishfaq et al., 2022). The generally accepted phenomenon is the cuticular route of nutrient entry (Oosterhuis, 2009). A typical nutrient absorption pathway through foliage involves foliar adsorption, followed by cuticular penetration, uptake and absorption into cellular compartments of leaf, followed by translocation and utilization. Another possible mechanism is through stomatal penetration through the process of diffusion along the stomatal pores (Fernández et al., 2013).

The efficacy of nutrient and biostimulants uptake in plants depends on the physicochemical properties of spray formulation such as pH, the surface tension of the spray liquids, and retention of spray fluid on the leaf surface. Additionally, the inherent nature of the spray formulation such as the molecular size of the nutrients or stimulatory compounds, ionic charge and solubility determines the success of penetration into the leaf (Pandey et al., 2013). FS is generally applied either through fertigation (addition of stimulants/nutrients in irrigation systems) process or through aerial sprays which are effective in improving nutrient use efficiency (Renuka et al., 2018). Foliar application of microalgae extracts has been proven successful in enhancing the growth of several crop plants. Cellular extracts of *Chlorella vulgaris*, *Spirulina platensis*, *Scenedesmus* spp., *Nostoc* spp., *Anabaena* spp., *Dunaliella salina* have been reported to enhance the growth, biomass yield (shoot weight), fruit yield, leaf pigment content, tolerance to abiotic stress such as drought, salinity and temperature stress in horticultural crops such as tomato (Gitau et al., 2022), lettuce (La Bella et al., 2021; Puglisi et al., 2022), capsicum (Elarroussia et al., 2016), onion (Gemin et al., 2022) and beans (Li et al., 2014; Elarroussia et al., 2016). In addition to the horticulture crops, FS of cellular extracts of *Spirulina platensis* and *Scenedesmus* spp., resulted in enhanced root weight, plant growth, number of flowers per plant, earliness of flowering and flower diameter in *Petunia* x hybrid plant (Plaza et al., 2018). Evaluation of the composition of microalgae cell hydrolysates revealed the presence of plant growth-promoting substances such as phytohormones (auxins and cytokinins), signaling molecules such as betaines, AAs, vitamins, polyamines, (spermine and spermidine), and polysaccharides mainly beta-glucan apart from micronutrients (González-Pérez et al., 2022). Among the various microalgae species, extracts and hydrolysates derived from *Spirulina platensis*, *Chlorella vulgaris* and *Scendesmus* spp., are most utilized for FS application (Arahou et al., 2022; González-Pérez et al., 2022).

### 3.3 Soil and root drench

Roots are the interface between soil and plant that sustain plant growth mainly by mobilizing nutrients, conducting external stimuli and initiating plant defence response to stressors while the soil is a finite nonrenewable resource that forms the basis of agriculture (Ma et al., 2022). Maintaining the health of roots and soil is essential for sustainable agriculture and this is achieved through the application of soil conditioners and fertilizers that replenish soil health and provide nutrients to roots. However, in intensive agricultural practices, the use of chemical fertilizers and conditioners has resulted in soil compaction, acidification, decreased fertility and imbalance of soil microflora aggravating soil diseases (Ye et al., 2020). This necessitates the use of biodegradable, less harmful soil conditioners and fertilizers. Soil drenching involves proportionate mixing of biofertilizers or stimulants during sowing that enhances plant growth. The mechanism of action of biostimulants/biofertilizers by soil drenching method is by agglomeration of soil particles with organic molecules such as polysaccharides, promotion of biological mineralization of complex nutrients and restoration of soil microflora (Karthikeyan et al., 2009; Alvarez et al., 2021).

Microalgae-based soil drenching applications involve the addition of live microalgae suspensions or dried biomass with suitable carrier materials for the application. Soil is the most economically viable carrier for agriculture applications. However, aerial contamination is the major issue with soil, necessitating alternative carrier materials. Agricultural and agro-industrial wastes such as bagasse, peat, wheat straw, vermiculite and animal manure are effective carriers in the application of cyanobacteria and microalgae in soil drenching applications (Renuka et al., 2018). Application of algae as biofertilizer in soil drenching is done as consortia of cyanobacteria/microalgae or as a consortium consisting of a combination of cyanobacteria and rhizobial bacterial species in a suitable carrier (Alvarez et al., 2021). As discussed earlier, the main application of these consortia is for soil amendment and crop productivity enhancement. The most commonly used cyanobacterial combination is *Anabaena* spp., and *Nostoc* spp., finding application in a variety of crops such as corn, rice, wheat, cotton and chickpea. The use of cyanobacterial consortium resulted in enhanced availability of soil N and P, increased soil enzyme activity such as nitrogenase, dehydrogenases, and proliferation of soil microflora leading to enhanced biomass and crop productivity (Karthikeyan et al., 2009; Prasanna et al., 2015a; Prasanna et al., 2015b). Further, a synergistic effect of cyanobacteria with rhizobial bacteria such as *Brevundimonas diminuta*, *Mesorhizobium ciceri*, *Azotobacter* spp., *Pseudomonas putida* etc., resulted in enhanced N_2_ fixation, P solubilization, soil micronutrient content and microbial activity (Alvarez et al., 2021). Several authors reported that the use of synergistic bacterial and cyanobacterial consortium in pot or field experiments resulted in the enhancement of macro and micronutrients in cereal crops such as rice (Rana et al., 2015). Similarly, there was an significant increase in the increased grain yield and leghaemoglobin content in nodules of chickpea (Prasanna et al., 2017). The beneficial effects were observed in flower crops such as chrysanthemum with increased flower diameter (Kanchan et al., 2019).

The major benefit of inoculating cyanobacteria was a reduction in the requirement for chemical NPK fertilizers. Most of the above-mentioned reports utilized live cyanobacteria or consortia partially replacing the chemical NPK fertilizers. The available reports uniformly suggest that the use of cyanobacterial consortia resulted in grain yield in crops such as corn and rice similar to chemical fertilizers albeit with a savings of N fertilizer to the tune of 50% (Prasanna et al., 2015a; Prasanna et al., 2015b; Prasanna et al., 2015c). In the case of green microalgae, the application of *Chlorella vulgaris* in synergy with *Pseudomonas putida* resulted in enhanced soil P mobilization to rice plants and prevented arsenic translocation in plant tissues suggesting the dual role of microalgae as biofertilizer and heavy metal sequestration (Srivastava et al., 2018). In the case of rice, treatment with cyanobacteria species such as *Anabaena variablis* and *Nostoc* spp., by root drench method enhanced the plant height, leaf length and grain yield compared to the inorganic fertilizers (Singh and Datta, 2007; Innok et al., 2009). Similarly, the use of eukaryotic microalgae biomass, specifically green algae *Chlorella* spp., enhanced the growth and yield of vegetable plants. For example, the application of *Chlorella pyrenoidosa* by root drench method enhanced the grain yield and shoot weight in soybean (Dubey and Dubey, 2010). Extracts of *Chlorella vulgaris* enhanced the growth of wheat, maize and tomato (Shaaban, 2001b; Coppens et al., 2016; Barone et al., 2018). The other microalgae species that have been reported to possess biofertilizer properties are *Scenedesmus* spp.*, Dunaliella* spp.*, Spirulina* spp.*, Scenedesmus quadricauda*, and *Nannochloropsis* spp. (Ammar et al., 2022). A summary of different modes of application of biostimulants derived from microalgae is presented in **Table 1**.

**Table 1:** Methods of application of microalgae biostimulants and their effect on plants.

Microalgae/extracts	Crop evaluated	Outcome(s)					References
		Enhanced germination rate & seedling vigor	Improved root parameters	Increased biomass yield	Enhanced nutritional content of seeds	Biotic/Abiotic stress tolerance	
Seed treatments							
*Spirulina platensis* (seed coating of Spirulina platensis extracts)	*Raphanus sativus*	**+**				**+**	(Godlewska et al., 2019)
*Spirulina* extract	• *Triticum aestivum* • *Hordeum vulgare*	**+**		**+**			(Akgül, 2019)
*Spirulina platensis* extract	*Calotropis procera* Ait	**+**	**+**			**+**	(Bahmani Jafarlou et al., 2021)
*Spirulina platensis* extract	*Vigna mungo* L.	**+**	**+**	**+**	**+**	**+**	(Thinh, 2021)
Phycocyanin extract [*Spirulina platensis*]	*Solenum lycopersicum* L.	**+**		**+**	**+**		(Metwally et al., 2022)
*Chlorella* spp. cell suspension	• *Triticum aestivum* • *Hordeum vulgare*	**+**	**+**	**+**			(Odgerel and Tserendulam, 2016)
*Chlorella vulgaris*	• *Solanum lycopersicum* L., • *Cucumus sativus*		**+**	**+**			(Bumandalai and Tserennadmid, 2019)
*Acutodesmus dimorphus* Live culture and extracts	*Solanum lycopersicum* var. *Roma*	**+**					(Garcia-Gonzalez and Sommerfeld, 2016)
*Nostoc commune aqueous extracts*	*Oryza sativa* L.	**+**	**+**	**+**			(Abedi Firoozjaei et al., 2021)
• *Dunaliella salina* extract • *Phaeodactylum tricornutum* extract	*Capsicum annuum* L.	**+**	**+**			**+**	. (Guzmán-Murillo et al., 2013)
Consortia of • *Microcystis aeruginosa* MKR 0105 • *Anabaena* spp. PCC 7120 • *Chlorella* spp.	*Zea mays* L.	**+**	**+**	**+**			(Grzesik and Romanowska-Duda, 2014)
• *Chlorella vulgaris* extract • *Scenedesmus quadricauda* extract	*Beta vulgaris*	**+**	**+**				(Puglisi et al., 2020)
Extracts of consortium • *Chlorella* spp., • *Scenedesmus* spp., • *Spirulina* spp., • *Synechocystis* spp.	*Solanum lycopersicum* L.	**+**	**+**	**+**			(Supraja et al., 2020a)
• *Chlorella vulgaris* • *Scenedesmus quadricauda*	*Beta vulgaris* L.		**+**				(Barone et al., 2018)
Consortia of • *Chlorococcum* spp. • *Micractinium* spp. • *Scenedesmus* spp. • *Chlorella* spp.	*Spinacia oleraceae*	**+**		**+**	**+**		(Rupawalla et al., 2022)

Soil/Root drenching							
Microalgae/ extracts	Crop evaluated	Improved root parameters	Enhanced biomass/crop yield	Enhanced leaf phytochemical content	Improved soil quality & soil enzyme activity	Biotic/Abiotic stress tolerance	References
*Spirulina platensis*	*Zea mays* L.		**+**		**+**		(Tuhy et al., 2015)
*Spirulina platensis* suspension	*Vicia faba* var. Giza 843	**+**	**+**			**+**	(Osman et al., 2016)
*Scenedesmus subspicatus*	*Allium cepa L*	**+**					(Gemin et al., 2022)
*Navicula Spp.*	• *Solanum lycopersicum* L., • *Capsicum annuum* L., • *Solanum melongena*		**+**		**+**		(Alshehrei et al., 2021)
Extract of *Spirulina maxima* and *Chlorella ellipsoida*	*Triticum aestivum*			**+**	**+**	**+**	(Abd El-Baky et al., 2010)
*Nostoc, Anabaena, Westiellopsis, Aulosira*, and Scytonema	*Oryza sativa* L.		**+**		**+**		(Paudel et al., 2012)
Extract of *Chlorella vulgaris* CCAP 211/11C	*Lactuca sativa* L.	**+**	**+**	**+**	**+**		(Puglisi et al., 2022)
*Polysaccharide extracts of Spirulina platensis, Dunaliella salina, porphyridium* spp.	*Solanum lycopersicum* L. var. Jana F1		**+**	**+**	**+**	**+**	(Rachidi et al., 2020)
*Scenedesmus obliquus Chlorella vulgaris and Anabaena oryzae* biomass	*Musa* spp.	**+**		**+**	**+**	**+**	(Hamouda and El-Ansary, 2017)
*Chlorella vulgaris* biomass with cow dung	*Solanum lycopersicum* L.	**+**		**+**	**+**	**+**	(Suchithra et al., 2022)
• Nannochloropsis • Consortia of *Ulothrix* spp. and *Klebsormidium* spp.	• *Solanum lycopersicon* cv., • *Solanum lycopersicum* var. Maxifort		**+**	**+**			(Coppens et al., 2016)

Foliar spray							
Microalgae/ extracts	Crop evaluated	Enhanced biomass/crop yield	Early flowering and increased enzymatic activity	Enhanced leaf pigment content & number of fruits/flowers	Improved nutritional quality of plants	Biotic/Abiotic stress tolerance	References
*Arthrospira spp*	*Beta vulgaris* L.	**+**			**+**		(de Oliveira et al., 2013)
Polysaccharides extract of *Spirulina platensis*	• *Solanum lycopersicum* L. • *Capsicum annuum* L.,	**+**			**+**		(Elarroussia et al., 2016)
*Spirulina* extract	*Cynara cadunculus* L.	**+**		**+**	**+**		(Amer et al., 2019)
*Spirulina* extract	*Zea mays* L.			**+**	**+**		(Kiran et al., 2020)
*Arthrospira fusiformis*	*Allium sativum*				**+**		(Shalaby and El-Ramady, 2014)
Cell extract of *Chlorella vulgaris*	*Triticum aestivum* L. var. Giza 69	**+**			**+**		(Shaaban, 2001a)
*Chlorella vulgaris* cell suspension	*Vicia faba*					**+**	(Li et al., 2014)
*Chlorella fusca*	• *Cucumis sativus* • *Arabidopsis thaliana*					**+**	(Kim et al., 2018; Lee et al., 2020)
*Chlorella vulgaris*	*Cyamopsis tetragonoloba* (L.) Taub.		**+**		**+**	**+**	(Kusvuran and Can, 2020)
*Chlorella vulgaris* extract	*Lactuca sativa*	**+**		**+**	**+**		(La Bella et al., 2021)
*Chlorella vulgaris*	*Brassica oleracea* var. italica		**+**	**+**		**+**	(Kusvuran, 2021)
*Chlorella vulgaris* extract	Lactuca sativa L.		**+**		**+**		(Puglisi et al., 2022)
*Scenedesmus* spp. extract	*Triticum aestivum* L. var. Gemmiza 9	**+**			**+**		(Shaaban et al., 2010)
*Dunaliella salina* exopolysaccharides	*Solanum lycopersicum* L.	**+**	**+**	**+**		**+**	(El Arroussi et al., 2018)
*Scenedesmus subspicatus*	*Allium cepa L*	**+**			**+**		(Gemin et al., 2022)
• *Acutodesmus dimorphus* extract	*Solanum lycopersicum* var. *Roma*	**+**	**+**		**+**		(Garcia-Gonzalez and Sommerfeld, 2016)
• *Scenedesmus* spp. extract • *Arthrospira platensis* cell hydrolysate	*Petunia x hybrida*	**+**	**+**		**+**		(Plaza et al., 2018)
• *Nostoc muscorum SOS14* extract • *Anabaena oryzae SOS13* extract	*Lactuca sativa* L.			**+**	**+**		(Mohsen, 2016)
Extracts of microalgae consortium • *Chlorella* spp., • *Scenedesmus* spp., • *Spirulina* spp., • *Synechocystis* spp	*Solanum lycopersicum* L.	**+**		**+**	**+**		(Supraja et al., 2020a)
Cell lysates of • *Chlamydomonas reinhardtii* CC124 • *Chlorella* sp.MACC360	*Solanum lycopersicum L*.	**+**	**+**	**+**			(Gitau et al., 2022)
Polysaccharides Extract of • *Dunaliella salina* MS002 and MS067 • *Phaeodactylum tricornotum* MS023, • *Porphyridium* spp. MS081, *Desmodesmus* spp. • *Spirulina platensis* MS001	*Solanum lycopersicum* L.				**+**	**+**	(Rachidi et al., 2021)
Polysaccharide extracts of • *Chlorella vulgaris* • *Chlorella Sorokiniana*	*Solanum lycopersicum* L.		**+**			**+**	(Farid et al., 2019)

+ indicated positive effects.

### 3.4 Effect of biostimulants and their method of applications on plant metabolism

As discussed earlier, the method of bio stimulant application is mainly dependent on the type of crop and the need of the crop, i.e., whether for nutrient enhancement or for tolerance against biotic and abiotic stresses. The method of biostimulant application and timing of application significantly affect the response of plants to stimulants. For example, in a study with lettuce, the response to *Chlorella vulgaris* extracts was different between root drenching and FS applications (Puglisi et al., 2022). In the case of root drenching, the microalgae extract exerted significant influence on carbon metabolism compared to a foliar application as observed with enhanced activities of malate dehydrogenase and citrate synthase, key enzymes involved in carbon fixation (Kreb’s cycle) supported with enhanced carbon content in the biomass and root and shoot weight (Puglisi et al., 2022). However, the N metabolism was influenced both by root drenching and FS as evidenced by increased activity of enzymes such as glutamate synthase and glutamine synthetase involved in N metabolism with both the treatments (Puglisi et al., 2022). This was morphologically validated with enhanced protein content in the shoot, increased leaf pigments and biomass weight. Similar observations of enhanced N metabolism and increased shoot and root N and biomass protein contents were observed with foliar application of *Scendesmus quadricauda* extracts in lettuce, commercial algae-based biostimulants on spinach (Fan et al., 2013; Ronga et al., 2019; Puglisi et al., 2020).

**Figure 2:**
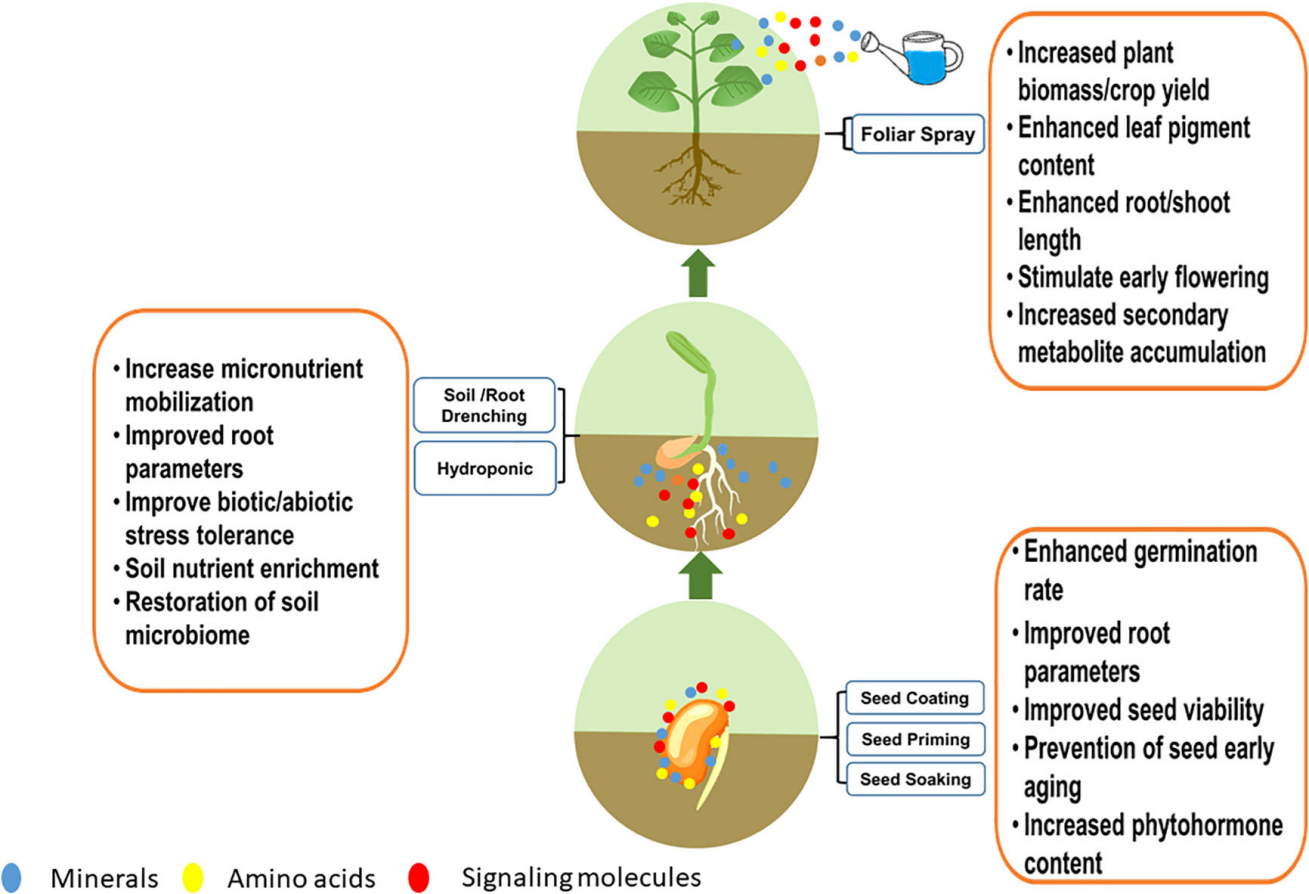
Methods of microalgae biostimulant application and their effects in plants.

In addition to supporting plant growth, bio stimulant application promotes stress tolerance in plants by means of activation of secondary metabolism. Several reports have been published with enhanced abiotic/biotic stress tolerance (**Table 1**). This observation can be attributed to the increased activity of the enzyme PAL involved in phenylpropanoid pathway involved in the synthesis of phenolics and flavonoids which function as plant defense molecules (Dehghanian et al., 2022). Microalgae extracts derived from *Chlorella vulgaris*, *Scenedesmus quadricauda* significantly enhanced the PAL activity in crops such as lettuce and sugar beet (Barone et al., 2018; Puglisi et al., 2020; Puglisi et al., 2022). Among the different methods of application, FS acted instantly in increasing the enzymatic activity compared to soil drenching. This was evidenced by time dependent expression of key enzymes (Puglisi et al., 2022). The PAL enzyme showed immediate increase in its activity after FS while the root drenched samples showed delayed expression of the enzymes at 4^th^ day indicating influence of application method on the time of physiological response (Puglisi et al., 2022). The immediate physiological response to FS as compared to root drenching applications could be attributed to the faster rate of absorption through stomata compared to root cells (Hong et al., 2021; Arahou et al., 2022). This has been earlier validated with nano-fertilizer applications of micronutrients where foliar application of minerals modulated plant growth better and faster compared to soil application (Alshaal and El-Ramady, 2017). For commercial and large scale applications, root drench and foliar applications seem to be best options of bio stimulant application. However, for faster physiological response in plants, FS of biostimulants is generally preferred. It is reiterated again that the mode of bio stimulant application is case dependent and varies with respect to the crops requirement. A summary of functions attributed with different methods of bio stimulant application is presented in **Figure 2**.

## 4 Microalgae in amelioration of abiotic stress in plants

Increasing human population and climate change exacerbated by anthropogenic activities has resulted in severe stress on the global crop production (Pereira, 2016). One of the significant impact of climate change is global warming resulting in a 0.3°C per decade mean increase in the global temperatures and a corresponding decrease in global crop productivity by -5% per °C (Lobell and Gourdji, 2012). Climate change and environmental conditions such as prolonged heat waves (higher atmospheric temperature), varying intensity of rainfall, failure of monsoons, and high CO_2_ concentration negatively affect plant physiology and consequently affect crop productivity (Ferguson, 2019; Chaudhry and Sidhu, 2021). This can be attributed to the altered photosynthetic and carbon assimilation mechanisms in plants in response to climate change (Reddy et al., 2010). Some of the major impacts of climate change on plant physiology, growth and development are (i) lower germination rate, (ii) decreased photosynthetic rate, (iii) reduction in shoot biomass and root/shoot length, (iv) root/shoot ratio imbalance, (v) poor stomatal conductance, (vi) reduced number of leaves, (vii) declined chlorophyll content, (viii) lower protein content, (ix) poor membrane stability (Chaudhry and Sidhu, 2021). The common underlying mechanism behind most of the negative impacts of climate change induced abiotic stress is through generation of ROS that alter protein synthesis, induce lipid peroxidation, alter membrane permeability, inactivate enzymes and degrade nucleic acids leading to cell death (Singh et al., 2019).

Plants have developed important adaptive strategies to counter climate change-induced abiotic stress such as *de novo* synthesis and regulation of phytohormones, accumulation of osmolytes, *de novo* synthesis of heat shock proteins, and enhanced activity of enzymatic antioxidants (Chaudhry and Sidhu, 2021). Phytohormones and signaling molecules play an important role in the upregulation of the abiotic stress defence mechanisms exhibited by plants. For example, drought stress in plants leads to enhancement in ABA levels, which in turn induces stomatal closure and regulates water balance in plants (Sah et al., 2016). Another mechanism to counter drought stress and salt stress is by an accumulation of osmolytes such as proline, trehalose, sucrose and glycine betaine. Accumulation of these molecules creates a negative osmotic potential inside the cell which leads to the entry of water into cells to regulate turgidity and maintain water balance and cellular osmolarity (Sharma et al., 2019). Similarly, under high temperature and salinity stress, JA and SA promote the expression of antioxidant enzymes like superoxide dismutase, APX, and CAT that scavenge ROS (Wang et al., 2020b).

**Figure 3:**
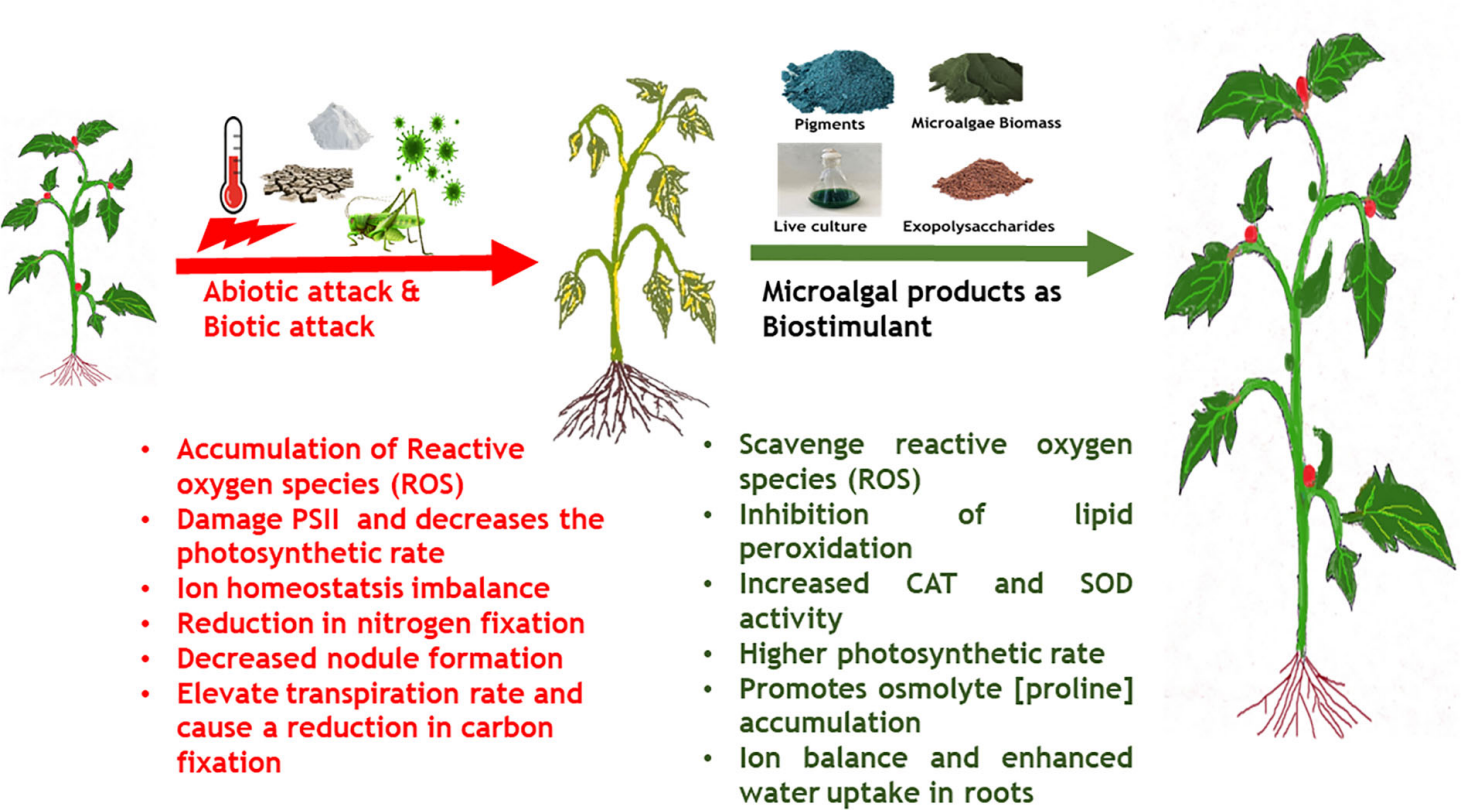
Effects of microalgae bio stimulants in biotic and abiotic stress tolerance in plants.

Use of microalgae and derived extracts as bio stimulant, especially towards mitigating abiotic stress has been in an upward trend in recent years. As described in earlier section, microalgae derived metabolites have wide range of functions such as signaling molecules, growth enhancers, antioxidant, natural mineral chelators that promote plant growth (Lee and Ryu, 2021). Among the various metabolites, EPS derived from both cyanobacteria and microalgae have shown plant immuno stimulatory properties along with other growth promoting activities in the rhizosphere regions such as mineral complexation, water retention etc. (Chanda et al., 2019). Application of sulfated polysaccharide extracts derived from *Dunaliella salina* enhanced the salinity tolerance in tomato and pepper plants by enhancing the antioxidant enzyme (POD, SOD, APX, CAT) activities (El Arroussi et al., 2018). In another study, polysaccharide extracts derived from *Spirulina platensis*, *Dunaliella salina*, *Porphyridium* spp., and *Phaeodacylum tricorntum* showed bio stimulatory effects when applied on tomato plants by enhancing PAL and chitinase enzyme, total polyphenol content, ROS scavenging activity and biosynthesis of very long chain fatty acids that constitute cuticular wax of leaves (Rachidi et al., 2020). Mutale-Joan et al. (2020) reported that injection of polysaccharides enriched liquid extracts of several microalgae species into tomato plants stimulated *de novo* lipid biosynthesis especially, palmitic and stearic acid involved in cuticular wax formation and linoleic acid involved in jasmonate pathway. The group further reported enhanced shoot, root and chlorophyll accumulation and correlated these observations to higher uptake of N and K in roots. Metabolomics’ analysis revealed that microalgal polysaccharides triggered accumulation of pyridine-3-carboxamide, (active amide form of vitamin B3) that promotes growth in plants. In another study by the group, a combined extract of microalgae and cyanobacteria such as *Dunaliella salina, Chlorella ellipsoidea, Aphanothece* spp.*, and Spirulina maxima* offered defense against salinity stress up to 150 mM NaCl in tomato plants by enhancing the activity of SOD and CAT, restoration of ion homeostasis by enhanced K^+^ uptake over Na^+^ ions and triggering of fatty acid degradation and conversion to alkane biosynthesis that constitute cuticular wax towards maintenance of water levels in hydric stressed plants. Further, it was observed that, application of combined microalgae-cyanobacteria extracts enhanced osmolytes accumulation, in this case proline (Mutale-Joan et al., 2020). A report by Kusvuran (2021) demonstrated that foliar application of *Chlorella vulgaris* extracts at 5% v/v strength alleviated drought stress induced oxidative stress in broccoli plants by enhancing the activities of CAT, SOD, APX, glutathione reductase and inhibiting lipid peroxidation. Further, *C. vulagris* extracts enhanced total chlorophyll, carotenoid, polyphenols and flavonoid content in plant tissues thereby promoting growth and secondary metabolite (non-enzymatic antioxidant defense response). The various abiotic stress tolerance imparted by microalgae derived extracts and bio stimulatory compounds are listed in **Table 2** and **Figure 3**.

**Table 2:** Microalgae mediated abiotic stress tolerance in plants.

Microalgae/consortia	Plant species	Abiotic factor	Observations	Mode of action	References
*Dunaliella salina* *Phaeodactylum tricornutim*	*Capsicum annuum* L.	Salinity stress [Up to 50mM NaCl]	• Increase in germination rate • Increase in shoot/root fresh weight • Resistance to salinity induced oxidative stress	• Scavenging superoxide free radicals • Inhibition of lipid peroxidation • Increased CAT and SOD activity • Ion balance and enhanced water uptake in roots	(Guzmán-Murillo et al., 2013)
*Dunaliella salina* *Chlorella ellipsoidea* *Aphanothece* spp., *Arthrospira maxima*	*Solanum lycopersicum* L. *Var. Jana F1* [tomato]	Salinity stress [Up to 150mM NaCl]	• Improved photosynthetic activity • Enhanced nutrient absorption • Increased shoot and root weight • Enhanced root volume • Larger leaf area • Improved osmotic adjustment	• Promotes osmolyte [proline] accumulation • Scavenging of ROS through increased CAT and SOD activity • Enhanced K^+^ uptake over Na^+^ • Restoration of ion homeostasis • Promoting fatty acid transformation to alkanes and cuticular wax biosynthesis	(Mutale-Joan et al., 2020)
*Dunliella salina* exopolysaccharides	*Solanum lycopersicum* L. *Var. Jana F1* [tomato]	Salinity stress [up to 6 gL^-1^]	• Increase in root and shoot length • Increase in total weight of the plant • Increased water holding capacity of roots	• Enhanced protein biosynthesis • Restoration of ion homeostasis • Enhanced K^+^ uptake over Na^+^ ions • Enhanced osmolyte accumulations [proline, glycine-betaine] • Enhanced alkane content in leaves with concomitant decrease in very long chain fatty acids	(El Arroussi et al., 2018)
*Nannochloropsis salina* *Chlorella vulgaris*	*Moringa oleifera* LAM	Salinity stress [up to 6gL^-1^]	• Enhancement of leaf area and leaf number • Improved photosynthetic activity • Enhanced stem and root DW • Enhanced polyphenol content [rutin and gallic acid] in leaves	• Enhanced K^+^ uptake over Na^+^ ions • Increase in non-enzymatic antioxidant defense systems [polyphenols]	(Al Dayel and El Sherif, 2021)
*Chlorella vulgaris* - [applied as live culture suspensions]	*Vicia faba* L. cv. Da qing pi	Drought stress	• Improved water use efficiency • Reduction in leaf transpiration rate	• Induction of stomatal closure • Reduction in stomatal aperture through ROS mediated signaling pathway • Promotes CAT activity and scavenges H_2_O_2_	(Li et al., 2014)
*Chlorella vulgaris* [applied as foliar spray]	*Brassica oleracea* var. *italica ‘Barokka’*	Drought stress	• Enhanced growth of the plants • Enhanced photosynthetic pigment content • Increased nutrient uptake	• Inhibition of lipid peroxidation • Mitigation of oxidative stress through ➢ Increased secondary metabolites like phenolic acids and flavonoids [non-enzymatic defense response] ➢ Increased antioxidant enzymes activity such as SOD, CAT, APX, GR	(Kusvuran, 2021)
*Oscillatoria agardhii* [Applied as nano silicon product]	Triticum spp., [Wheat]	Drought stress	• Increase in grain yield • Grain flour protein content	• Increase in the activity of hydrolytic and defense enzymes (CAT, SOD, APX)	(Haggag et al., 2018)
General biostimulant properties exhibited by microalgae					
*Chlorella vulgaris Scenedesmus quadricauda* extracts	*Beta vulgaris* L.	Biostimulant effect	Enhanced root growth characterized by • Increased root length • Increased number of root tips • Increased fine root length • Increased root surface area	• Upregulation of genes involved in biosynthetic pathway of primary and secondary metabolism • Upregulation of intracellular transport • Upregulation of nutrient acquisition genes such as inorganic phosphate transporter	(Barone et al., 2018)
*Arthrospira platensis*, *Dunaliella salina, Porphyridium* spp. [Polysaccharides extract]	*Solanum lycopersicum* L. *Var. Jana F1*	Biostimulant and growth enhancement effect	• Increased shoot length, weight and shoot nodes number • Increased photosynthetic pigments content	• Increased activity of N assimilatory enzymes - nitrate reductase & NAD-GDH activities • Enhancement of sterols/steroidal glycol-alkaloids in plants • Increased alkanes content in leaves suggesting cuticle wax formation • Enhanced protein biosynthesis	(Rachidi et al., 2020)
*Chlorella vulgaris* [Applied as FS]	*Vigna mungo* L.	Biostimulant and growth enhancement effect	• Increase number of root nodules • Increased root and shoot length • Increased root and shoot weight • Increased photosynthetic pigment content in plants • Increased number of pods, seeds and weight of seeds	Enhanced nutrient uptake through mineral solubilzation and mobilization into roots	(Dineshkumar et al., 2019)
**Cyanobacteria** - *Anabaena oryzae* *Anabaena doliolum* *Phormidium fragile* *Caothrixx geitonos* *Hapalosiphon intricatus* *Aulosira fertilissima* *Tolypothrix tenuis* *Oscillatoria acuta* *Plectonema boryanum* [Applied as Live cultures]	*Oryza sativa Var UPR 1823*	Plant growth promotion and stress tolerance in rice plants	• Increased root and shoot length • Increased plant fresh weight • Increased photosynthetic pigment content • Increased metabolite content in leaves [phenolics]	• Root colonization and N fixation • Increased antioxidant enzymes activity - peroxidase and PAL • Increased phytohormone – IAA & IBA concentration in leaves promoting plant growth	(Singh et al., 2011)

ROS, Reactive Oxygen Species; SOD, Superoxide Dismutase; APX, Ascorbate Peroxidase; CAT, Catalase; GR, Glutathione Reductase; NAD-GDH, nicotinamide adenine dinucleotide glutamate dehydrogenase; PAL, Phenylalanine Ammonia Lyase; IAA, Indole-3-acetic acid; IBA, Indole-3-butyric acid.

## 5 Microalgae as biopesticides

Biopesticides are naturally occurring organisms or biologically derived compounds that suppress the growth and proliferation of plant pathogens ranging from bacteria, fungi, insects and nematodes (Fenibo et al., 2021). Bio pesticides are broadly two types viz., microbial and biochemical; and microalgae derived bio pesticides fall under both these categories. Microalgal biopesticides act by inhibiting the growth, development and reproduction of plant pathogens or by competitively inhibiting the nutrients to the pathogen restricting their growth (Costa et al., 2019). Among the various species of algae, cyanobacteria have found significant applications as biopesticides. The most commonly used cyanobacteria are *Anabeana* spp., *Nostoc* spp., *Spirulina platensis, Oscillatoria* spp., *and Tolypothrix* spp., (Costa et al., 2019; Hernández-Fernández et al., 2021). Along with these cyanobacteria, chlorophycean microalgae such as *Chlorella vulgaris*, *Chlorella fusca, Scendesmus* spp., have been attributed with biopesticide activity (Bileva, 2013).

### 5.1 Anti-microbial properties of cyanobacteria and microalgae

Cyanobacteria are known to secrete EPS and anti-microbial compounds that inhibit the growth of plant pathogens. *Nostoc* spp., *Nodularia harveyana*, produce cyclic compounds such as 4,4’ –dihydroxybiphenyl, norharmane and diterpenoids that show anti-bacterial activity (Volk and Furkert, 2006). Norharmane is indole alkaloids mostly secreted by cyanobacterial species belonging to the order Nostacales that have mutagenic properties and inhibit key metabolic enzymes such as monoamine oxidase, indoleamine 2,3-dioxygenase, nitric oxide synthase (Volk, 2008). These antimicrobials were effective against several fungal pathogens. For example, Abdel-Hafez et al. (2015) reported the fungicidal activity of extracts obtained from *Nostoc muscorum* and *Oscillatoria* against *Alternaria porri* that causes a purple blotch of onion. The group analyzed the fungicidal extracts revealing the presence of inhibitory compounds such as β-ionone, norharmane, and α-isomethyl ionone. Along with these, a variety of antimicrobial compounds such as nostocyclyne A, nostocin A, ambigol A and B, hapalindoles and scytophycins, tjipanazoles, fischerellin-A have been identified and applied as biocontrol agents obtained from cyanobacteria such as *Nostoc* spp., *Scytonema* spp., *Tolypothrix* spp., *Fischerella* spp. respectively (Singh et al., 2016). These antimicrobials inhibit protein synthesis, enzymatic functions and cell division in pathogens. In another mechanism, some anti-microbial compounds especially siderophore type molecules competitively bind to nutrients such as Fe, Cu and deprive the nutrients to pathogens as observed in the case of biocidal activities of *Pseudomonas* spp., (Lee and Ryu, 2021). Further, cyanobacteria such as *Anabaena laxa*, *Anabaena variabilis*, *Nostoc* spp., *Calothrix elenkinii* produce hydrolytic enzymes, mainly cell wall-degrading enzymes such as chitosanase, β-1,4-glucanase, and β-1,3-glucanase that hydrolyse the cell wall of pathogens such as *Pythium aphanidermatum* (Prasanna et al., 2008; Gupta et al., 2011; Natarajan et al., 2013). Apart from directly acting on fungal pathogens, microalgal and cyanobacterial extracts enhance plant defence mechanisms by enhancing the activity of hydrolytic enzymes (endochitinases such as β-N-acetylhexosamindase, chitin-1,4-β-chitobiosidase, Endoglucanase – β-1,3 glucanase), polyphenol oxidase and POD (Roberti et al., 2015). Further, cyanobacterial extracts enhance the activity of PAL enzyme that catalyzes phenylpropanoid pathway involved in biosynthesis of SA attributed to systemic resistance to infections in plants and phenolics and phytoalexins (Singh et al., 2011; Babu et al., 2015). Application of cyanobacterial biomass and cell-free extracts to the soil resulted in reduction in disease symptoms. For example, Chaudhary et al. (2012) and Prasanna et al. (2013) reported that biopesticide formulation of *Anabaena* spp., led to a 10% to 15% reduction in damping-off disease in tomato seedlings and significant increase in plant growth when challenged with pathogenic fungi. Similarly, the application of *Spirulina platensis* resulted in a reduction of root rot, root wilt and damping off symptoms in Moringa plant (Imara et al., 2021).

### 5.2 Insecticidal properties of cyanobacteria and microalgae

In addition to the antimicrobial properties, microalgae possess insecticidal properties, specifically nematicidal properties owing to the secretion of neurotoxins such as anatoxin-A, microcystin, nodularins inhibiting the growth of nematode pests. Anatoxin-A, mimics neurotransmitter acetylcholine and bind irreversibly to acetylcholine receptors leading to continuous muscle contraction in nematode pests leading to their immobility (Mankiewicz et al., 2003). Number of cyanobacterial species such as *Oscillatoria chlorina*, *Aulosira fertilissima, Spirulina platensis, Amphora cofeaeformis* have been reported to exhibit nematicidal properties against soil borne nematodes such as root knot nematode (*Meloidogyne arenaria, Meloidogyne incognita), Xiphinema index* that affect the root formation (Khan et al., 2007; Chandel, 2009; Bileva, 2013; El-Eslamboly et al., 2019). Root knot nematodes forms gall like structure in the roots leading to hardening of root surface ultimately affecting nutrient uptake in plants. Soil application of cyanobacteria and microalgae such as *Chlorella vulgaris* inhibited the nematode growth and reduced the gall formation in roots and promoted root and shoot growth in plants (Khan et al., 2007). Microalgae derived secondary metabolites such as hexamethyl phenol, methoxyphenyl and flavonoids were shown to inhibit nematode growth and reduction of gall formation in roots of *Cucumis sativus* plants and concomitant enhancement in fruit yield and quality (El-Eslamboly et al., 2019). Additionally, cyanobacteria such as *Anabaena* spp., and *Scytonema* spp., produce peptide toxins that act as repellents to insect pests. (Sathiyamoorthy and Shanmugasundaram, 1996) reported presence of low molecular weight (<12 kDa) peptides in *Scytonema* spp., that have strong odour repelling cotton leaf chewing insects such as *Helicoverpa armigera* and *Stylepta derogate*. Similarly, Abdel-Rahim and Hamed (2013) reported insecticidal properties of extracts obtained from *Anabaena flos aquae* against *Spodoptera littoralis* larvae (Cotton leaf worm) that destroys cotton crops. The authors reported that the cyanobacterial extracts suppressed the oviposition in the insects and caused sterility in insects. A list of bio pesticide and crop protection properties of cyanobacteria and microalgae is presented in **Table 3** and the mode of action is presented in **Figure 3**.

**Table 3:** Biotic stress tolerances imparted by microalgae.

Microalgae/consortia	Host plant species	Biopesticide activity	Mode of action	References	
Anti-fungal activity					
*Nostoc* strain ATCC 53789	*Solanum lycopersicum* L.	• Fungicidal activity against *Sclerotinia sclerotiorum*, • Concentration dependent fungicidal activity • Enhanced root and shoot length	High adventitious root formation increasing nutrient uptake in the plants	(Biondi et al., 2004)	
*Anabaena, Oscillatoria, Nostoc, Nodularia* and *Calothrix* species	NR	Fungicidal activity against *Alternaria alternate, Botrysis cinerea, Rhizopus stolonifer* *Phytopthora capsici*, fusarium oxysporium, *Colletotrichum gleosporoides*	• Reduced fungal colonization in plant tissues • Enhancement of plant defense mechanisms mainly activity of hydrolytic enzymes - PPO, chitinase, and PAL • Contains depsipeptide named cryptophycin 1 which promotes antiproliferative and anti-mitotic activity • Glycosylated lipopeptides – hassallidins	(Kim et al., 2006; Kim and Kim, 2008; Vestola et al., 2013)	
*Nostoc commune* FA-103	*Solanum lycopersicum* L.	• Fungicidal activity against f. sp. *Lycopersici* • Minimum inhibitory concentration – 150 µg per seed • Reduction in number of infected seedlings • Increase in root, stem length • Increase in number of hairy roots			
*Anabaena variabilis* RPAN59 and *Anabaena oscillarioides* RPAN69	*Solanum lycopersicum* L	• Fungicidal activity against *Pythium debaryanum, Fusarium oxysporum lycopersici, Fusarium moniliforme and Rhizoctonia solani* • Increase in the number of leaves • Enhancement of plant height and weight • Reduction in disease severity	Fungicidal activity due to presence of hydrolytic activity of cyanobacterial extracts	(Chaudhary et al., 2012)	
*Anabaena laxa* and *Calothrix elenkinii*	*Coriandrum sativum* L. *Var*. NRCSS-ACr1, *Cuminum cyminum* L. Var. RZ-209, *Foeniculum vulgare* Mill. *Var*. NRCSS-AF-1	• Fungicidal activity against *Fusarium oxysporum* ITCC 95 and *Macrophomina phaseolina* ITCC 5141 • Enhancement in germination rate and soil chlorophyll • Increase in root and shoot length • Improvement in the vigour index of seed	• Enhancement of plant defense mechanisms mainly activity of hydrolytic enzymes - PPO, chitinase, • Increased activity of endogluconase and peroxidase in plants	(Kumar et al., 2013)	
*Nostoc muscorum* and *Oscillatoria* spp.	*Allium cepa* L.	• Fungicidal activity against *Alternaria porri* that causes Purple blotch of Onion • Disease reduction by 55.1% – 66.5%	• Phenolic acids, alkaloids derived from cyanobacterial extracellular filtrates induce fungicidal activity • Major fungicidal compounds include - β-ionone, norharmane, and α-isomethyl ionone	(Abdel-Hafez et al., 2015)	
Consortia of cyanobacteria [*Calothrix elenkinii* and *Anabaena laxa* RPAN8] and *Trichoderma* spp.,	*Gossypium hirsutum* F1861 and Gossypium arboreum CISA 310	• Fungicidal activity against Rhizoctonia spp. • Root colonization by cyanobacteria • Increased N fixation • Enhancement of soil N and P • Increased fresh and DW of plant	• Enhanced defense enzyme activity such as of β-1,3 endoglucanase, β-1,4 endoglucanase and chitosanase in plants • Enhanced activity of stress defense enzymes such as peroxidase and PAL	(Babu et al., 2015)	
*Anabaena* spp., BEA0300B strain	*Cucurbita pepo* [Zucchini plants cv. Consul]	Fungicidal activity against *Podosphaera xanthii* (Castagne) U. Braun and Shishkoff 25.4% reduction of the infected leaf area	Fungicidal activity attributed to increased activity of ➢ Endochitinases such as β-N acetylhexosamindase, chitin-1,4- β-chitobiosidase, ➢ Endoglucanase – β-1,3 glucanase and peroxidase	(Roberti et al., 2015)	
Phycobiliproteins from *Spirulina platensis* and *Hydropuntia cornea*	*Lycopersicum esculentum* L	• Fungicidal activity against *Botrytis cinerea* • Inhibited mycelial growth and spore germination • Reduced fruit disease incidence	• Antioxidant property of phycobilins attributed to fungicidal activity	(Righini et al., 2020)	
*Spirulina platensis*	*Moringa oleifera* LAM	• Fungicidal activity against *Sclerotium rolfsii*, *Fusarium oxysporum*, *Fusarium solani*, *Rhizoctonia solani* • Controlling Damping-off, Root Rot and Wilt Diseases • Increased shoot weight and growth of plant • Enriched nutrients of Moringa leaf	NR	(Imara et al., 2021)	
Insecticidal activity					
*Anabaena cyliindrica, Anabaena oyzae, Nostoc muscorum, Tolypothrix tenuis*	*Oryzae sativa* var. Indica IR 28 *[short duration]*	• Insecticidal activity against *Chilo agamemnon* [stem borer] and *Hydrellia prosternalis* [leaf miner] • Increased grain yield and weight • Reduction in use of chemical N fertilizer	NR	(Yanni and Abdallah, 1990)	
*Anabaena flos aqua*	*In vitro* studies	• Insecticidal activity against *Spodoptera littoralis* (Boisd.) larvae [Cotton leaf worm] • Reduction in inset pupation • Reduction in fertility of eggs	• Cyanobacterial extracts suppressed oviposition of the adults • Induced sterility and reduced fecundity in insects	(Abdel-Rahim and Hamed, 2013)	
*Oscillatoria chlorina* [Cyanobacterial biomass applied to the soil]	*Lycopersicum esculentum* L	• Nematicidal activity against *Meloidogyne arenaria* [root knot nematode] • Reducing the number of galls in the root • Increased vegetative growth and root mass production	• Potential nematicidal activity due to presence of cyanobacterial neurotoxins – Anatoxin-a • Additionally, secondary metabolites such as polyketides, cyclic peptides, lipopeptides induce toxicity in nematodes	(Khan et al., 2007)	
*Chlorella vulgaris*	*Vitis vinifera* grapevine cv. Palieri	Nematicidal activity against root ectoparasite *Xiphinemia index* Increase in root and shoot length Enhancement of fresh and dry weight of plant	NR	(Bileva, 2013)	
*Spirulina platensis* *Amphora cofeaeformis*	*Cucumis sativus* L*, Hesham hybrid F1*	• Nematicidal activity against *Meloidogyne incognita* [root knot nematode] • Reduction in the egg production • Reducing the number of galls in the root • Increased host plant vegetative growth, fruit quality and yield	Microalgae derived secondary metabolites such as asetamide and phenolic compounds such as hexamethyl phenol, methoxyphenyl phenolic acids, flavonoids act as nematicides	(El-Eslamboly et al., 2019)	
*Aulosira fertilissima*	*In vitro* studies	• Nematicidal activity against Meloidogyne triticoryzae and Meloidogyne incognita • Inhibition of the hatching of root knot nematode	Extracellular secretions such as polysaccharides induce nematicidal activity	(Chandel, 2009)	

NR, Not reported; PPO, Polyphenol Oxidase; PAL, Phenylalanine Ammonia Lyase.

Review of literature clearly indicates that microalgae and cyanobacteria have excellent plant growth promoting properties. However, their utilization in large scale has not seen the commercial light. Some of the important challenges associated with commercial utilization of microalgae in agro-chemicals sector are high cost of cultivation, varying biomass productivities, high cost and energy intensive downstream processes in obtaining purified metabolites demonstrating plant growth promoting applications (Sharma et al., 2013; Smetana et al., 2017). This warrants identification of processes and technologies that reduce the cost of biomass production and improve energy efficiency in downstream processes. Following section describes the various steps involved in microalgae biomass generation and discuss strategies for low cost biomass production and multi product utilization to achieve environmental sustainability along with economic viability.

## 6 Production of microalgae based bio fertilizers and biostimulants

### 6.1 Microalgae biomass production

Microalgae have been considered as a potential industrial source of food, chemicals and bio-products owing to their higher photosynthetic efficiency and high unit dry matter per yield/land area in comparison to land plants (Chen et al., 2022). The presence of myriad of chemical compounds comes from their ability to adapt to wide environmental conditions and adopt different modes of nutrition such as autotrophic (completely photosynthetic), mixotrophic (ability to utilize both reduced organic carbon and light sources) and heterotrophic (fermentative approach with utilization of organic carbon source) (Udayan et al., 2022). Microalgae biomass is the functional material and the primary basis for biofertilizer or biostimulants production. Systematic evaluation of biomass production processes and understanding their suitability is very critical in mass production of biofertilizers and agro-chemicals. A typical microalgae biomass production or derived products involves three major steps, *viz*., (i) cultivation, (ii) harvesting and (iii) downstream processing consisting dewatering, extraction, and purification or formulation. The production strategies vary with respect to intended application such as for food, feed, fuel or agriculture applications. Microalgae are generally mass cultivated either in open raceway ponds (ORP) or closed PBRs utilizing the phototrophic mode of nutrition exploiting solar energy, carbon dioxide and inorganic minerals. Amongst the two systems, ORP are commercially preferred over PBRs owing to their low capital and operational costs while PBRs are preferred for accurate control of culture parameters and production of high value products such as proteins, pigments (carotenoids and xanthophylls), and polyunsaturated fatty acids (Udayan et al., 2022). The choice of cultivation depends on the species/strains and further downstream processes selected. Although ORP is widely preferred, major disadvantages include contamination of culture, low biomass productivity and susceptibility to predators and environmental conditions (Tan et al., 2020). Photo bioreactors offer better control the over the cultures with high biomass productivity, reduced contamination risks and higher metabolite yield; however, the major disadvantages are high capital investment, higher energy requirements in circulating culture and maintenance of temperature and risk of bio fouling and increased dissolved oxygen in cultures leading to cell death (Tan et al., 2020; Udayan et al., 2021; Udayan et al., 2022).

The second important step in the biomass production process is harvesting, which involves the separation of microalgae cells from the growth medium and the recycling of the spent medium. A typical microalgae culture has asolids content of less than 1%, (approximately 0.6 to 1 g biomass per litre culture) and needs to be concentrated between 15% and 25% solids content for further downstream processing (Milledge and Heaven, 2013). Several processes such as chemical -induced flocculation, bio-flocculation (coagulation induced by bacterial/fungal species), electrocoagulation, flotation, and membrane filtration have been developed and evaluated for harvesting microalgae biomass (Milledge and Heaven, 2013; Vandamme et al., 2013; Barros et al., 2015; Udayan et al., 2022). Despite the availability of enormous literature on the harvesting of microalgae, it is a general consensus that harvesting is the major bottleneck in the commercial production of microalgae biomass. This could be attributed to the wide range of cellular properties such as size, shape, the surface charge of microalgae cells necessitating customized solutions limiting commercial scalability (Kumar et al., 2022a). However, commercially a combination of flocculation followed by pressure filtration or centrifugation is considered economically viable with lower energy inputs (less than 0.1 kWh·kg^−1^ algae) (Fasaei et al., 2018). The success of a harvesting process is entirely dependent on the cultivation step where the cell density of the culture determines the energy expenditure incurred during harvesting. Cultivation in ORP is generally performed at low cell densities to avoid the shading effect and consequent dark fermentation at the bottom of the ponds (Shekh et al., 2022). This necessitates the deployment of multiple harvesting steps such as two-stage process of initial concentration to 2% to 3% solids content followed by dewatering to 15% solids content (Milledge and Heaven, 2013). However, this problem can be avoided with microalgae cultivation in flat plate PBRs at higher cell density reducing the energy and cost requirements during the harvesting stage (Milledge and Heaven, 2013).

The final step involved in typical microalgae biomass production is dewatering and downstream processing for further production of commercial products. Dehydration is the most energy-intensive process and has been a major area of concern and a limiting factor in the commercialization of microalgae products. The choice of dehydration is dependent on the end product application and earlier reports indicate that convective air drying of harvested biomass paste is the most economical option for large-scale production (Sirohi et al., 2022). However, it has been observed that heating of biomass beyond 70°C resulted in the loss of heat-labile compounds such as phycobiliproteins, antioxidants and secondary metabolites (Phong et al., 2017). For the biofertilizer application, mainly as a source of macro and micronutrients, the dewatering process does not influence biomass stability. However, for use as biostimulants such as phytohormone-rich extracts or pesticidal extracts, dehydration plays a crucial role as many of these compounds are heat sensitive necessitating optimization of dewatering and further downstream processes.

### 6.2 Life cycle analysis and sustainability of microalgae biomass production

In order to select a particular cultivation, harvesting and downstream strategy, a thorough evaluation of sustainability aspects involved in various stages of biomass production is required. For commodity products such as food, animal feed, biofuel or biofertilizers the choice of cultivation method must be cheaper with minimal energy expenditure. However, the choice of process varies between species/strains necessitating a detailed sustainability analysis. This study is known as life cycle analysis (LCA). A typical LCA involves assessment of process against six parameters namely global warming potential expressed as g CO_2_ eq. per kg biomass, acidification potential expressed as g SO_2_ eq. per kg biomass, eutrophication potential expressed as g PO_4_^-^ eq. per kg biomass, cumulative energy demand expressed as MJ per kg biomass, land use expressed as m^2^ per kg biomass and water usage expressed as L per kg biomass. These parameters could also be tested against other functional units that are potentially generated from microalgae biomass such as energy produced (in Kcal) in case of biofuels, or proteins/polysaccharides/pigments/or metabolite of interest (expressed in grams/kg biomass) (Kumar et al., 2022a).

Life cycle analysis of microalgae based products have been studied for various scenarios such as biofuels (Huang et al., 2022; Kim et al., 2022), food and nutraceuticals (Schade and Meier, 2020; Schade et al., 2020), multiproduct refineries (Arashiro et al., 2022; Ubando et al., 2022) biofertilizers (de Siqueira Castro et al., 2020; Arashiro et al., 2022). The major consensus among most of these studies is that the cultivation of microalgae in ORP is cheaper and less energy intensive compared to PBRs. However, the biomass productivity is roughly 12 times higher with PBRs (1.5 kg dry biomass m^-3^ day^-1^) compared to ORP (0.117 kg dry biomass m^-3^ day^-1^) (Silva et al., 2015). The lower sustainability quotient with respect to PBRs based cultivation could be attributed to the high capital costs involved in construction of PBRs which contribute to almost 85% of the total energy consumed in the process (Silva et al., 2015). Calculation of net energy returns, a simple ratio of energy produced over energy consumed for various cultivation steps indicated that biomass production using horizontal tubular PBRs had NER <1 while biomass produced with ORP or flat plate PBRs had NER >1, suggesting commercial feasibility of ORP based biomass production (Jorquera et al., 2010).

**Figure 4:**
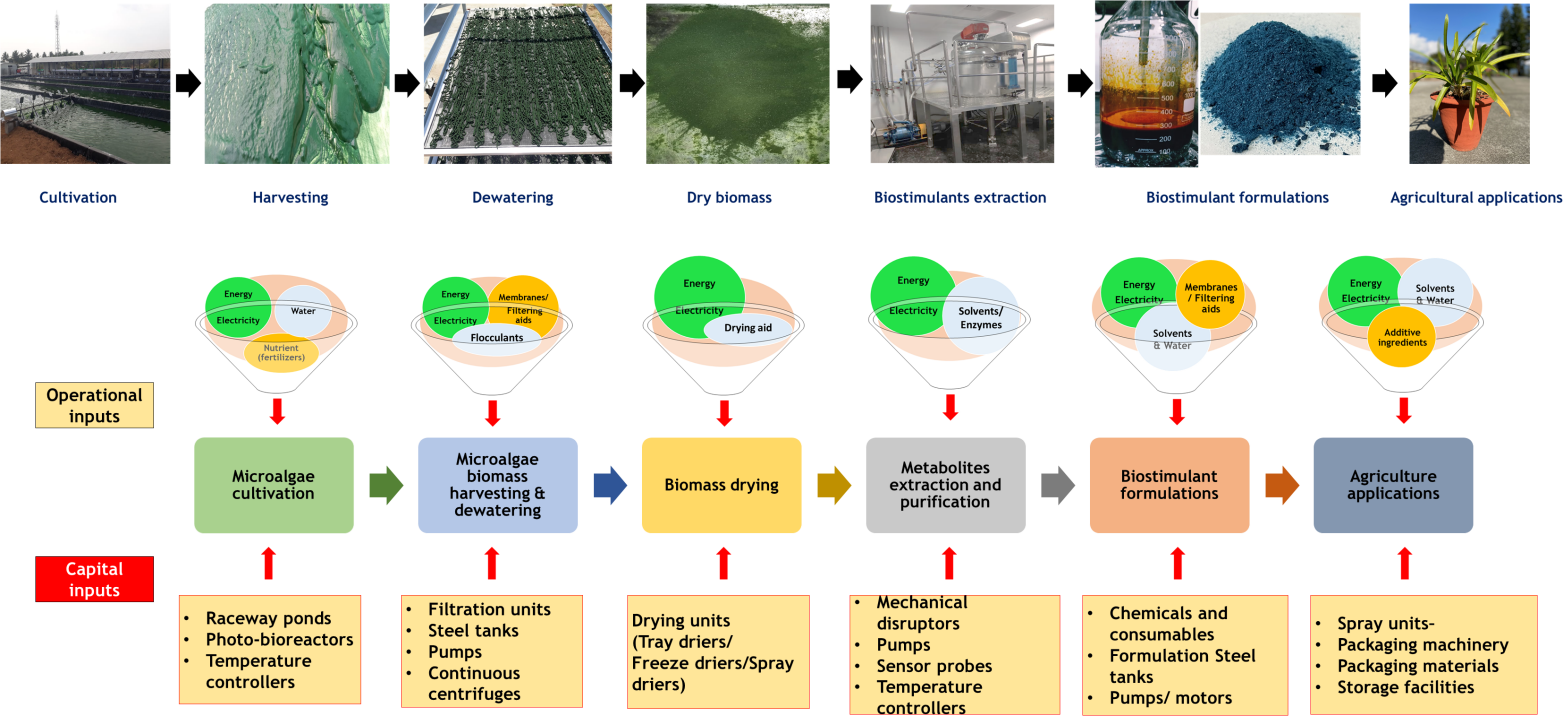
Energy inputs involved in microalgae biomass production towards biostimulants application.

Energy required for heating cultures in ORP and the energy required for injecting CO_2_ and maintaining the flow of cultures in PBRs and supply of reduced organic carbon sources in mixotrophic or heterotrophic cultivation are some critical factors that negatively influence microalgae based processes (Ighalo et al., 2022). Microalgae based processes had relatively lower land and water use compared to aquaculture processes, however, in comparison to poultry and insect farming the water and nutrient requirements were relatively higher for microalgae biomass production (Maiolo et al., 2020; Schade et al., 2020). The overall energy requirements for production of one metric ton (1 MT) of microalgae biomass was estimated between 12000 and 22000 kWh, which was 4 to 7 folds higher compared to poultry or insect meal based protein production (Maiolo et al., 2020). The capital and operational inputs contributing to the energy consumption as part of the life cycle assessment during microalgae cultivation and biomass processing is presented in **Figure 4**.

Water and nutrient requirements are the two critical parameters that determine the economics of microalgae biomass production. For example, the estimated nutrient requirement (inorganic salts) for the production of 1 MT of fresh biomass of microalga *Tetraselmis suecia* was 90 kg and the water requirement was approximately 15000 m^3^ per m^2^ per month, which is 10 times higher compared to other poultry and insect meal production (Maiolo et al., 2020). This high water requirement makes microalgae biomass-based products and processes unsustainable, necessitating the identification of cheaper sources of water and nutrients. Wastewater obtained from industrial processes could be a potential source of nutrient-rich water for the cultivation of microalgae (Dagnaisser et al., 2022). The concept of utilization of industrial wastewater for the cultivation of microalgae has been practised for quite some time now with several success stories and enormous literature (Carraro et al., 2022; Nagarajan et al., 2022; Satya et al., 2022; Yan et al., 2022). Microalgae have the unique ability to grow in non-potable waters and have excellent bioremediation potential with high removal efficiencies against nitrates, phosphates, and ammonia in waste streams and reduce the chemical (COD) and biological oxygen demand (BOD) of wastewaters (Ahmed et al., 2022; Díaz et al., 2022). Further, the ability to use industrial flue gas as a source of CO_2_ and the presence of carbon concentration mechanism in microalgae make them an attractive resource for dual applications of bioremediation and metabolite/biomass production (Çakirsoy et al., 2022; Ighalo et al., 2022; Ma et al., 2022).

### 6.3 Dual application of bioremediation and biofertilizer application

The dual application of bioremediation and microalgae biomass production has been successfully exploited previously for biofuel production (Ali et al., 2022; Das et al., 2022). The concept has helped to improve the economics of biomass production with microalgae having the ability to grow in different types of industrial wastewater streams such as that of steel slag, cement, food processing, distillery, aquaculture, leather tannery and anaerobic digestates (You et al., 2021; Rambabu et al., 2022; Venugopal, 2022). As discussed earlier, microalgae consistently showed a nutrient removal efficiency against nitrates, phosphates, organic carbon, ammonia and other nutrients above 70%, and in a few cases above 90% from the initial levels (Abdelfattah et al., 2022; Nagarajan et al., 2022). The ability of microalgae to absorb nutrients in abundance could be attributed to the special physiological mechanisms such as carbon concentration mechanism, lipid remodeling and P allocation in fast-growing microalgae as described earlier (Çakirsoy et al., 2022). Microalgae prefer ammonia over inorganic N sources as their assimilation is less energy intensive and can be directly incorporated into AAs and other N moieties (Sarma et al., 2021). This provides an opportunity for ammonia-rich wastewaters such as agriculture run- offs, agro-industrial residues and anaerobic digestates to act as an excellent N sources in microalgae cultivation (Wang et al., 2018).

A typical microalgae biomass contains relatively higher nutrient content compared to other organic fertilizers, especially N and P. The average N content ranges between 4.9% and 7.1% N while the P content ranges from 1.5% to 2.1% (Khan et al., 2019). Further with the presence of a luxury uptake mechanism, microalgae accumulate nutrients from the surrounding medium and act as a concentrated source of nutrients for plant growth. The use of wastewater-derived microalgae biomass as biofertilizers and source of biostimulants has been a rising trend with several case studies being published in recent years (Álvarez-González et al., 2022; Arashiro et al., 2022; Dagnaisser et al., 2022). **Table 4** lists various case studies available on the applications of wastewater-generated biomass as biofertilizer. Domestic and municipal wastewater has been predominantly used as a source of nutrients for microalgae biomass production (**Table 4**). However, the major disadvantage of this is the varying levels of nutrients as frequent nutrient composition changes affect biomass productivity. Among the various wastewater resources evaluated, food industry effluents are promising for the recovery of various algae-derived bioproducts owing to their better quality compared to other effluents (Arashiro et al., 2022). Further, the group reported that microalgae biomass production utilizing wastewater significantly reduced the energy expenditure and environmental impact thus enhancing the sustainability of the whole process. Further integration of industrial gases, specifically CO_2_-rich flue gases along with wastewater utilization enhances biomass productivity and bioproducts recovery (Yadav et al., 2019; Carraro et al., 2022).

**Table 4:** Wastewater mediated microalgae biomass production towards biofertilizer and biostimulant applications.

Wastewater source	Microalgae used	Nutrient removal efficiency	Method of Application	Crop(s) evaluated	Observation	References
Domestic waste water	*Chlorella* spp.	Selenium (Sodium selenite) - 44% TSS - 86% COD – 70% TC - 67% TP - 77% NH_4_^+^ - 93%	• Foliar spray • Soil drenching • Seed treatment	*Phaseolus vulgaris*	• Enhanced germination rate • Selenium enrichment in seeds and leaves [bio-fortification]	(Li et al., 2021)
Domestic waste water	Consortia of *Chlorella* spp.*, and Scenedesmus* spp.	Nitrates - 96% NH_4_^+^ - 98% PO4^3-^ – 95% COD- 83% TOC - 86% TN - 94%	Deoiled algal biomass as biofertlizer	*Solanum lycopersicum*	• Enhanced shoot and root weight • Enhanced macro [N, P, K] and micronutrients [Ca, Mg, Fe] in biomass • Increased tomato yields	(Silambarasan et al., 2021a)
Aquaculture wastewater	*Spirulina platensis*	Nitrates – 50% NH_4_^+^ - 95%	• Soil drenching [admixtures of soil and dry biomass] • Seed soaking	• *Eruca sativa* • *Ameranthus gangeticus*, • *Brassica rapa ssp. Chinensis*	• Enhanced germination rate • Increased seedling vigor • Enhanced plant height and root length • Increased biomass yield • Increased chlorophyll content	(Wuang et al., 2016)
Domestic waste water	*Scenedesmus* spp.	COD- 69% TIN - 91% TP - 81% NH_4_^+^ - 95%	Soil drenching [admixtures of microalga biomass paste and substrate]	*Ocimum basilicum L.*	• Enhanced leaf weight • Increased magnesium content in leaves • Overall performance similar to inorganic fertilizers	(Álvarez-González et al., 2022)
Sewage waste water	*Chlorella minutissima*	TDS - 96% TP - 70% K - 45% NH_4_^+^ - 90% BOD - >90% COD – 80% Nitrates – 89%	Soil drenching [admixtures of dry microalga biomass and soil]	• *Spinacia oleraceae* • *Zea mays*	• Enhanced available N and organic carbon in soil • Enhanced biomass and root yield • Increased soil enzymes activity [urease, nitrate reductase & dehydrogenase]	(Sharma et al., 2021)
Brewery effluents	*Scenedesmus* *obliquus*	COD - 71% TN - 88% TP - 30% NH_4_^+^ - 81%	• Algal cell suspension • Deoiled biomass fertilizers	• *Triticum aestivum* • *Hordeum vulgare*	Increased the germination and sprouting	(Ferreira et al., 2019)
Piggery waste water	• *Tetradesmus obliquus* • *Chlorella protothecoides* • *Chlorella vulgaris* • *Synechocystis* spp., • *Neochloris oleoabundans* • *Nostoc* spp.,	COD - 62-79% NH_4_^+^ - 79-92% PO_4_^3-^ - 90 -98%	Seed treatments with algae biomass	• *Solanum lycopersicum* • *Cucumis sativus* • *Hordeum vulgare* • *Glycine max* • *Nasturium officinale* • *Triticum aestivum*	• Increased germination index • Increased root length in wheat and cucumber • Biopesticidal activity against *Fusarium oxysporum*	(Ferreira et al., 2021)
Dairy effluent	*Chlorella pyrenoidosa*	BOD - 88% COD - 85% NH4^+^ - 99% PO4^3-^ − - 97%	Encapsulated algal biomass as biofertilizers	*Oryza sativa*	• Enhanced the length of root and shoot in rice seedlings	(Yadavalli and Heggers, 2013)
Paddy soaked rice mill waste water	*Chlorella pyrenoidosa*	NH4^+^ - 69.4% PO4^3-^ – 64.7%	• Seed treatment with microalgae cell suspensions • Soil drenching with live algae biomass	*Abelmoschus angulosus*	• Increased germination percentage • Increase number of leaves • Increased plant height	(Umamaheswari and Shanthakumar, 2021)

TSS, Total Suspended Solids; TDS, Total Dissolved Solids; TOC, Total Organic Carbon; TC, Total Carbon; BOD, Biological Oxygen Demand; COD, Chemical Oxygen Demand; TN, Total nitrogen; TP, Total phosphorous; TIN, Total Inorganic nitrogen.

### 6.4 Challenges associated with wastewater algae as biofertilizers

Although microalgae show prolific growth and efficiently remediate nutrients in waste effluents, the process of utilizing wastewater treatment derived biomass for agriculture applications has certain inherent challenges. These include scalability of biomass production, presence of xenobiotic residues and heavy metals in biomass and contamination of cultures with bacteria, fungi and virus limiting their widespread application (Abdelfattah et al., 2022; Amin et al., 2022). The first critical challenge in wastewater mediated biomass production is meeting the light requirement for cultures. The industrial effluents derived from food, livestock, aquaculture processing and sewage have high suspended solids, reducing the light penetration into the growth medium consequently affecting photosynthetic rate and biomass productivity (Abdelfattah et al., 2022). This light penetration issue is a major challenge in scalability of wastewater mediated large scale biomass production. The average biomass yield obtained utilizing wastewater ranged between 0.7 g L^-1^ to 1.4 g L^-1^ with microalgae such as *Scendesmsus* spp., *Chlorella* spp., *Desmodesmus* spp., *Tetradesmus* spp., *Selenastrum* spp., predominantly effective in effluent treatment (Rani et al., 2021; Amin et al., 2022). Further the biomass productivity significantly varies with respect to the temperature, pH, effluent nature and nutrient content (Rani et al., 2021). A “one shoe size fits all” approach cannot be applied with respect to biomass production utilizing industrial effluents, thus requiring a case specific standardization and cultivation strategy. The second critical issue is harvesting and dewatering of microalgae from effluent treatment ponds and reactors. As discussed earlier the biomass concentration is very low in large scale algae cultures requiring energy intensive dewatering steps.

Apart from scalability, microalgae cultivated utilizing dye and tannery effluents, and pharmaceutical wastes pose the challenge of presence of xenobiotic residues and heavy metals in the biomass. Microalgae have the ability to degrade organic hydrocarbons such as phenols, azo dyes, and benzene compounds such as naphthalene, antibiotics and hormones residues present in wastewater (Xiong et al., 2021; Touliabah et al., 2022). The mechanisms behind the degradation of organic compounds by microalgae include (i) secretion of extracellular polymeric substances such as polysaccharides, proteins and humic acid that degrade organic molecules, (ii) bioaccumulation of organic compounds followed by intracellular biotransformation through redox reactions, hydrolysis and conjugation with macromolecules and (iii) photolysis (Xiong et al., 2021). A range of antibiotics belonging to classes such as tetracyclines, cephalosporins, macrolides, penicillins, and beta-lactams have been degraded using microalgae with varied efficiency (Chandel et al., 2022; Wang et al., 2022). Similarly, microalgae remove heavy metals from effluents through the phenomenon of bio adsorption and bioaccumulation followed by chelation and vacuolar sequestration or biotransformation utilizing special intracellular chelating agents called ‘phytochelatins’ (Chugh et al., 2022). Although the ability of microalgae to remediate xenobiotics, heavy metals have been demonstrated, there are no pilot scale studies involved on the additive toxicity of microalgae biomass enriched with a heavy metal or a bio transformed molecules. On the other hand, high concentrations of heavy metals or xenobiotics, colored dyes have shown to induce oxidative stress in microalgae causing cell death (Bhatt et al., 2022). This defeats the whole purpose of microalgae mediated bioremediation. Further the coexistence of fungi, bacteria and viruses in the waste effluents pose another challenge in utilization of microalgae biomass. Microalgae and other microbes have been known to interact both mutually and competitively in a polluted environment such as industry effluents and sewage. The interactions have been supported through nutritional reciprocity, chemotaxis, communication through volatile organic compounds and signal transduction between microalgae and microbiome present in the wastewater environment (Ashraf et al., 2022; Astafyeva et al., 2022; Chegukrishnamurthi et al., 2022).

In addition to heavy metals and xenobiotics, co-occurrence of virulent microbes along with microalgae biomass and their consequent application as biofertilizers poses the risk of introduction of virulence into the ecosystem. Further occurrence of horizontal gene transfers between micro biome and microalgae in polluted waters have been demonstrated offering special adaptive mechanisms to microalgae such as tolerance to heavy metals and impart extremophilic properties (You et al., 2021). Though HGTs offer niche adaptive benefits to algae, it poses the risk of transfer of antibiotic resistance genes, disrupting existing transcription mechanisms of biotransformation thereby affecting the bioremediation process and consequently the biomass application as biofertilizer. The major drawback in deployment of microalgae biomass produced from wastewaters and effluents for biofertilizer application is the lack of pilot scale/industrial scale trials and understand the real time effect of environment on microalgae biomass production and its impact on the biomass productivity. Most of the published literature are based on lab scale trials that do not truly reflect real time scenarios necessitating systematic studies. Nevertheless, the concept of biomass generation through bioremediation of wastewater and industrial effluents offers an exciting opportunity in increasing the sustainability of microalgae biomass production; since microalgae biomass is the primary basis of supplying nutrients and stimulants for plant growth.

## 7 Microalgae based bio refineries and circular economy for sustainable agriculture

It has been advocated recently that the sustainability and commercial feasibility of the microalgae-mediated technologies come only from multiproduct utilization, *i.e*, through a biorefinery approach (Mehariya et al., 2021). Although means for producing microalgae biomass at low costs, utilizing wastewater and CO_2_-rich flue gas have been worked out, the high cost of downstream processing creates a net negative energy balance making the process unfeasible for commodity applications such as that of animal feed, agrochemicals and industrial chemicals (fuels) (Subhash et al., 2022). With newer findings on the potential of microalgae biomass and its metabolites for agricultural applications, it is imperative to identify sustainable biomass utilization routes for commercial feasibility. In this context, a biorefinery approach of complete valorization of microalgae biomass coupled with a circular economy route of reuse, recycling and refurbishing with minimal energy footprint could be effective in achieving the commercial feasibility of microalgae-derived agrochemicals (Geissdoerfer et al., 2017; Behera et al., 2021). In the case of microalgae-based circular economy, a typical model would be integrating wastewater treatment coupled with biomass production followed by downstream processing of the biomass for value-added products and utilizing the residual biomass obtained thereafter for food, feed, agriculture or bioenergy applications (Kholssi et al., 2021).

### 7.1 Microalgae bio refineries

**Figure 5:**
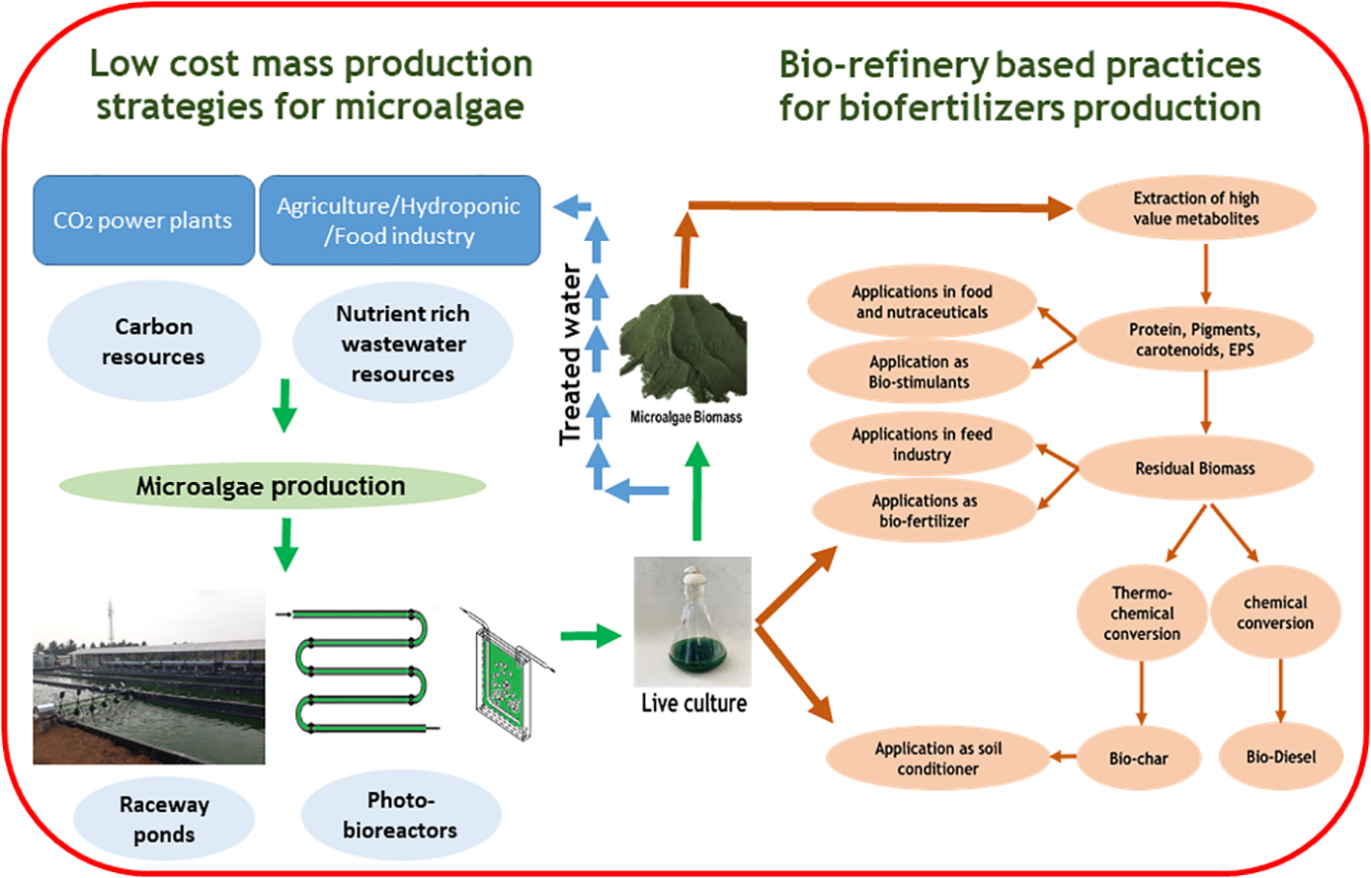
A typical microalgae biorefinery scheme.

Microalgae bio refineries involve complete fractionation and utilization of biomass analogous to petrochemical refinery (Vermuë et al., 2018). As discussed earlier, a typical microalgae based ingredient [food/feed/fuel] production would involve cultivation, harvesting, dewatering, extraction and purification steps involving multiple unit processes that are energy and cost intensive. To achieve commercial feasibility, prudent selection of processes followed by generation of high value ingredients is essential. Among the various commodities that can be produced from microalgae, biofuels are supposed to be cheapest. However, with the high process costs, the commercial feasibility of microalgae derived biofuels is questionable. To offset this uncertainty, several scenarios of multiproduct generation and biomass valorization strategies are being suggested such as (i) production of animal feed after biofuel extraction, (ii) gasification of deoiled biomass for generation of syngas, (iii) liquefaction of deoiled biomass for production of bio crude/bio char, (iv) anaerobic digestion of biomass to produce methane, (v) fermentation of deoiled microalgae biomass for bioethanol, (vi) application of deoiled biomass as biofertilizers and soil conditioners (Katiyar et al., 2021). For further economic feasibility and reduced energy footprints, integration of bio refinery with bioremediation of wastewaters and CO_2_ could enhance the commercial prospects along with environmental benefits. A typical microalgae biorefinery involving multiproduct utilization is presented in **Figure 5**.

Experimental trials have indicated positive outcomes from integrated biorefinery based routes towards biofertilizers application. Nayak et al. (2019) reported the use of deoiled *Scenedesmus* spp., biomass cultivated using domestic wastewater and coal-fired flue gas [2.5% v/v] as biofertilizers in the cultivation of rice. Deoiled *Scenedesmus* spp., biomass contained an entire spectrum of AAs and carbohydrates that promoted plant growth and reduced the requirements for chemical fertilizers. Similarly, *Chlorella minutissima* cultivated using sewage water was used as biofertilizer in field experiments for the cultivation of spinach and baby corn (Sharma et al., 2021). Phycoremediation with *Chlorella minutissima* resulted in a significant reduction (up to 80%) of both BOD and COD, and >50% of ammonia, nitrate and phosphate levels in the sewage water. The NPK content of microalgae biomass was higher compared to other natural manures such as vermicomposting, oil cake and biogas slurry. Application of microalgae biomass resulted in enhanced soil microbial activity, total organic carbon, available N and P as compared to soils enriched with chemical fertilizers (Sharma et al., 2021). Microalgae biomass obtained from external CO_2_ supplementation from flue gas showed enhanced nutrient content and exhibited biofertilizer potential. Sinha et al. (2021) reported that deoiled biomass of CO_2_ tolerant strain *Tetradesmus obliquus* CT02 exhibited biofertilizer properties when evaluated upon tomato plants. The deoiled biomass enhanced the germination percentage and index of tomato seedlings in comparison with commercial NPK fertilizers. In similar lines, cellular extracts of deoiled biomass of *Nostoc* spp. cultivated using municipal wastewater showed biostimulatory effects and improved the growth of lettuce plants. Foliar application of extracts derived from deoiled *Nostoc* spp., LS04 resulted in enhanced shoot and root length, fresh weight, DW, leaf chlorophyll and nutrient content compared to control groups (Silambarasan et al., 2021b).

### 7.2 Microalgae biochar for agricultural applications

In addition to the direct utilization of microalgae deoiled biomass and its extracts, bio char obtained through thermochemical conversion processes (hydrothermal carbonization, pyrolysis and Torre faction) of deoiled biomass could be used for agriculture applications (Mona et al., 2021). This integrated bio refinery route typically involves four steps *viz*., (i) cultivation of microalgae using wastewater; (ii) harvesting and downstream processing of biomass for lipid/hydrocarbon extraction; (iii) thermochemical conversion of deoiled biomass for bio char production and (iv) biofertilizer application of microalgae based bio char. Previous reports on use of bio char obtained from various agro-residues and biomass like straw, coffee husk, bamboo and variety of wood have been reviewed and reported to possess biofertilizer properties (Wang et al., 2020a). Application of bio char obtained from these biomasses in agro-forestry systems have resulted in enhanced soil fertility, improved soil texture especially in sandy and loamy soils through enhanced water retention, improvement in soil cation exchange capacities (CEC) leading to increased soil pH, and nutrient availability for plants in the rhizosphere region (Wang et al., 2020a). Thermochemical conversion of biomass results in oxidation of aromatic carbon groups and other complex organic molecules resulting in availability of carboxyl, carbonyl and other negatively charged functional groups that form complexes with cations thus enhancing the CEC of soil leading to nutrient retention (Laird et al., 2010). Further the porous microstructure and higher surface area of bio char causes slow release of nutrients to the plants thereby acting as slow release fertilizers reducing the problem of leaching and surface runoff (Gwenzi et al., 2015; Yu et al., 2019; Wang et al., 2020a). Thermochemical conversion of deoiled microalgae biomass could yield up to 90% bio char with higher nutritive value compared to deoiled microalgae biomass. Ultimate analysis of bio char and deoiled biomass revealed that thermochemical conversion enhances the net N, carbon, C/N ratio in bio char over the raw biomass (Phusunti et al., 2017). However, this depends on the thermos-conversion process parameters such as pyrolysis temperature and residence time during the process (Gan et al., 2018). The ultimate elemental analysis of biochar obtained from *Chlorella* spp. and *Nannochloropsis* spp., consisted 33% -50% w/w carbon, 33% - 57% w/w oxygen, 1% -5% w/w hydrogen and 4%-12% w/w N along with minerals such as Ca, Fe, Mg and K. The chemical composition of biochar, especially the mineralized N content indicated their suitability as biofertilizers especially for nutrient enrichment of soil (Borges et al., 2014; Chang et al., 2015). Biochar obtained from *Scenedesmus abundans* biomass cultivated using domestic wastewater had bio fertilization properties. The biochar contained 54.23% w/w carbon, 9.8% w/w hydrogen, 4.2% w/w ammoniacal N, 2.7% w/w sulphur and 28% w/w oxygen (Arun et al., 2020). The authors reported that biochar obtained from wastewater grown *Scendemsus abundans* biomass enhanced the growth, leaf and shoot weight and leaf chlorophyll content in tomato plants compared to chemical fertilizers. Apart from enhancing and mobilizing nutrient content in soil, microalgae derived biochar have been found to improve soil enzyme activity and promote soil microflora and soil microbial carbon content (Palanisamy et al., 2017). In another case study, use of algal biochar promoted defense response to drought stress in maize plants under deficit irrigation conditions. It was observed that algal biochar in consortia with plant growth promoting rhizobacteria *Serratia odorifera* accelerated plant growth by enhancing the nutrient availability in soil and enhancing photosynthetic activity (Ullah et al., 2020).

### 7.3 Integration of hydroponics and microalgae cultivation towards circular economy

Along with the use of municipal and sewage wastewaters, microalgae biomass obtained from wastewater generated from hi-tech agriculture systems such as hydroponics could be utilized used for biofertilizer application in a closed-loop model. Soilless cultivation such as hydroponics hasgained significant attention in recent years as the system can achieve maximum yield under a shorter time duration with excellent quality (Richa et al., 2020). Hydroponics utilizes nutrient solutions consisting of fertilizer salts in a definite ratio as per plant’s growth requirements. Typically, the nutrient solutions used for the cultivation of vegetables and other horticulture plants have electrical conductivity in the range between 0.8 and 2.5 dS m^-1^ and pH between 5 and 7 and are generally prepared using salts that have high solubility in water (Hosseinzadeh et al., 2017). The physicochemical properties of nutrient solutions used in hydroponic systems meet the essential nutrient requirements of microalgae suggesting its potential in microalgae cultivation (Zhang et al., 2017). Even the wastewater (hydroponic system drainage) solution obtained after a plant cultivation cycle contains high concentrations of residual N (150–600 mg L^-1^) and P (30–100 mg L^-1^) along with other nutrients such as sulphates, K, Ca, trace minerals and organic substances like humic acids and root exudates (Saxena and Bassi, 2013). This wastewater solution that when released into the environment results in eutrophication and imbalance in the aquatic ecosystem (Richa et al., 2020). Nutrient recycling of hydroponics wastewater using membrane filtration technologies [reverse osmosis, ultrafiltration], sand filtration, UV treatment and denitrification using biological filters, and chemical treatment has been generally practised (Patel et al., 2020; Rufí-Salís et al., 2020). However, challenges exist in wastewater recycling treatments as organic residues and root exudates present in wastewater require multiple strategies as they promote microbial growth (Richa et al., 2020).

**Figure 6:**
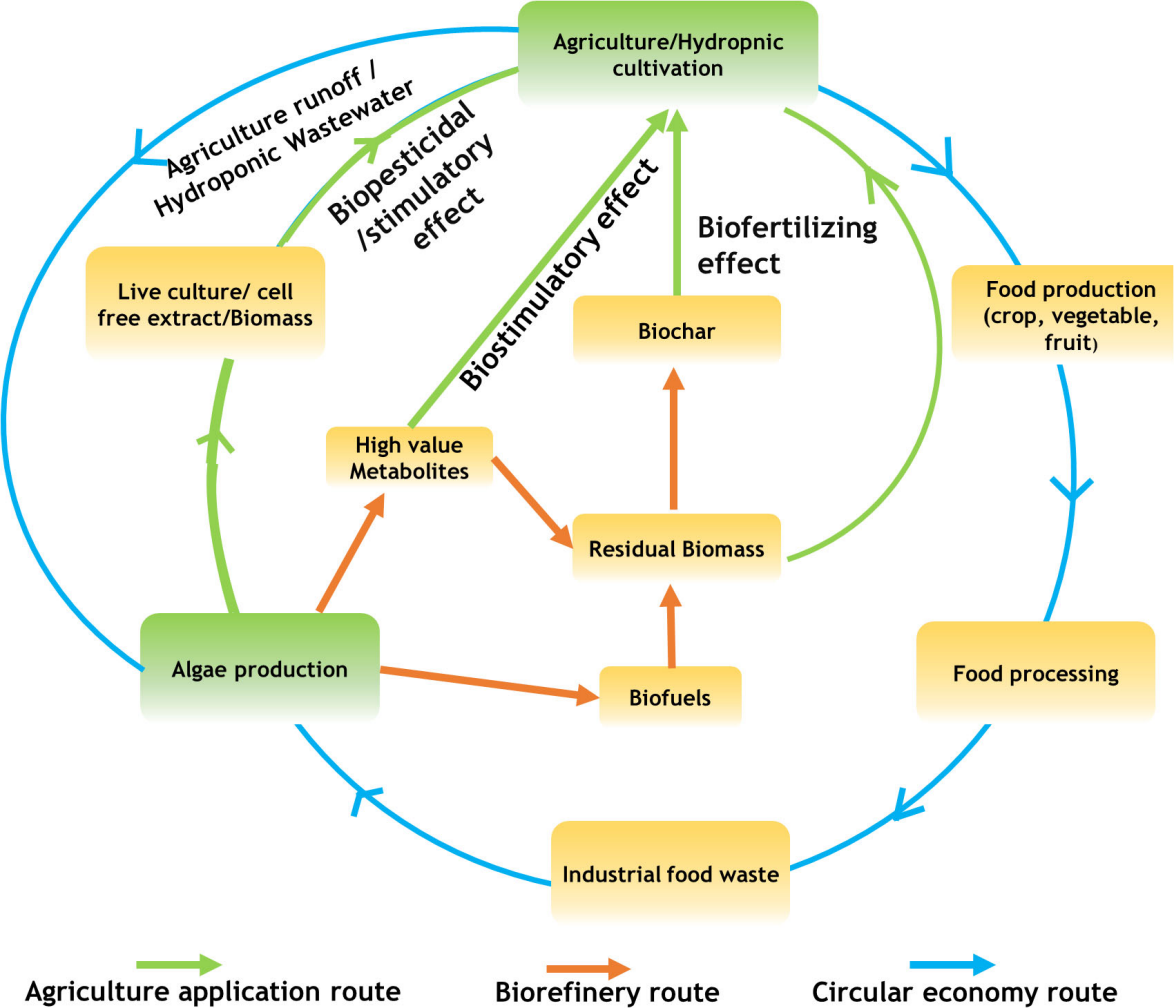
A typical closed loop circular economy model of microalgae integrated biorefinery coupled with biofertilizer and biostimulatory applications.

Hydroponics wastewater treatment and nutrient recycling could be effectively achieved by microalgae co-cultivation strategies. Co-cultivation of microalgae such as *Chlorella vulgaris* and *Scenedesmus quadricauda* with tomato plants in hydroponic system resulted in enhanced growth of both the plant and algae with biostimulatory properties exhibited by microalgae. This could be attributed to the mutual benefits exchanged by plant and algae where the roots exudates and inorganic nutrients promote algae growth and algal exudates induce stimulatory effects on to the plants (Barone et al., 2019). Zhang et al. (2017) reported that co-cultivation of microalgae *Chlorella infusionum* and tomato plants in hydroponic systems resulted in higher microalgae and crop biomass productivities when compared to individual monocultures. The enhancement in biomass productivity could be attributed to the aeration induced by microalgal photosynthesis and simultaneous utilization of CO_2_ from crop root respiration. Further co-cultivation reduces nutrient load during the drainage of the hydroponic wastewater. Supraja et al. (2020b) observed the above-mentioned phenomena of enhanced plant and microalgae biomass productivities and reduced the nutrient load in drain water during co-cultivation of microalgae consortia consisting *Chlorella* spp., *Scenedesmus* spp., *Synechocystis* spp., and *Spirulina* spp., with tomato plants. Co-cultivation resulted in the utilization > 80% of initial N, P and K in the nutrient media. All the aforesaid studies suggest that microalgae co-cultivation in hydroponics is a cost-effective solution for simultaneous microalgae biomass production and wastewater treatment. A typical closed loop circular economy model of microalgae integrated biorefinery is presented in **Figure 6**.

## 8 Microalgae based agro-chemicals: Market trends and way forward

Use of microalgae biomass and derived extracts as bio-fertilizers and growth stimulants have gained significant global attention in the recent past. Although, role of cyanobacteria and few microalgae species in N fixation and plant growth promotion have been known for many decades; the discovery of new applications of microalgae metabolites in agriculture and need for biorefinery approach in microalgae biomass utilization have renewed the interests of microalgae as alternative to synthetic agrochemicals (Kapoore et al., 2021; Lee and Ryu, 2021; Sharma et al., 2021). Analysis of the patent landscape of microalgae utilization in agriculture showed specific applications in plant growth promotion, plant protection (disease control), weed management and post-harvest quality improvement (Murata et al., 2021). The current global market for plant growth promoters and bio-stimulants is valued at USD 3.2 billion and forecasted to reach USD 5.6 billion by the year 2026 (https://www.marketsandmarkets.com/Market-Reports/biostimulant-market-1081.html). The demand for microalgae based fertilizers and plant growth stimulants was valued at USD 9479 thousand in 2021 with a compounded annual growth of 7.7% and is expected to grow to 8.7% by 2031. The market survey indicated that *Spirulina platensis*, *Chlorella vulgaris* and *Dunaliella salina* cumulatively contributed to 27.5% of microalgae based offerings to the fertilizers sector. [https://www.futuremarketinsights.com/reports/microalgae-fertilizers-sector]. As discussed in previous sections, microalgae biomass and extracts rich in polysaccharides, AAs have been commercially used for biofertilizer/bio stimulant applications. The bio-stimulant activity from microalgae have been demonstrated through different modes of applications such as (i) amendment of soil with algal formulations with suitable carriers, (ii) amendment of soil with algal dry biomass or suspended liquid culture, (iii) foliar spray of microalgae extracts and cell free supernatant, (iv) substrate or soil drench with alga culture (Chiaiese et al., 2018). Among the various application methods, FS was found to be the most effective (González-Pérez et al., 2022).

Despite the availability of enormous literature, commercial realization of microalgal technologies for agrochemical sector is still far, with several challenges to be overcome which are discussed below. Most critical challenges are survival, colonization and interaction of microalgae/cyanobacteria with the host plant. Challenges of lab to field transitions are critical as many good strains/consortia of strains with excellent performance *in vitro* perform very poorly under field conditions (Mitter et al., 2021). Some of the challenges involved in field trials are edaphic factors such as negative biotic interactions with resident microbiomes of host plant leading to competition and antagonism and high variability in soil physico-chemical properties like pH, moisture, temperature and salinity (de Siqueira Castro et al., 2017; Schütz et al., 2018). Apart from these, host plant acceptance and variability in plant growth [plant age] and its physiological status affect the functionality of biofertilizer or bio stimulant (Dennis et al., 2010). Thirdly, viability and stability of microbial formulations, in our case microalgal/cyanobacteria, under field conditions, persistence to varying temperatures and salinity are some critical parameters that affect the widespread use of biofertilizers (Kaminsky et al., 2019). Finally, practical challenges such as cost effectiveness, scalability and farmer’s acceptance determine the success of the technology.

Some potential solutions to overcome the aforesaid challenges are (i) developing a consortia of biofertilizers with wide ecological adaptations with broad pH, thermo, psychro and osmotic tolerances, (ii) isolation of strains from the host rhizosphere and native soil, (iii) personalized biofertilizers specific to soil or a crop, (iv) biofilm forming organisms for enhanced stability, (v) formulations and carriers that are stable to environmental fluctuations and use of technology such as encapsulation and milder dehydration processes that retain the bioactivity of the formulations (Mitter et al., 2021). Apart from plant growth enhancement, human biosafety of cyanobacterial and microalgal biofertilzers are essential as many cyanobacterial strains secrete toxins such as microcystins, nodularin’s and analchalins which affect neurological functions in humans and pose a significant risk (Kubickova et al., 2019). Currently, majority of commercial biofertilizers formulations consist of N_2_-fixing organisms such as *Actinorhizobium* spp., *Azospirillum* spp., *Azotobacter* spp., and *Rhizobium* spp. that have low health risk and has history of safe applications (Sessitsch et al., 2019). Therefore, it becomes imperative to evaluate the toxicology aspects of novel algae based biofertilizers in experimental animal models before its widespread use. A list of commercially marketed microalgae derived biostimulants and plant growth promoters are listed in **Table 5**.

**Table 5:** List of commercially marketed microalgae based bio-stimulants and plant growth promoters.

Marketed product	Company	Microalgae species	Claim Application	References
Algafert	Biorizon Biotech	Spirulina	• Growth and fattening of fruits. • Faster crop rooting • Natural color stimulation in fruits. • Size and homogeneous ripening. • Improving resistance against pests, thermal stress and disease.	https://www.biorizon.es/bioenhancers/hydrolyzed/algafert/?lang=en
Agrialgae organic orginal	AlgaEnergy	Microalgae	**• **High quality agricultural bio-stimulant based on an optimized combination of microalgae • High assimilation by the plant, promoting both the root and the foliage development of the plant • Increases its photosynthetic capacity and promotes the regeneration of damaged tissues • provides the crops with the tools to reinforce its immune system, making it more resistant to or boosting the recovery from stress episodes	https://www.agrialgae.es/tienda-online-bioestimulantes/agrialgae-organic-en/agrialgae-organic-original-2/?lang=en
Agrialgae premium			• AgriAlgae^®^ Premium includes biostimulants specifically designed to provide your crop with what it needs in each of the phenological phases of development. • Provides solutions for different stages like flowering, fruit setting, ripening and fattening, rooting, sprouting and stress	https://www.agrialgae.es/bioestimulantes-agricolas/agrialgae-premium-en/?lang=en
Blue Green Algae Biofertilizer	AlgEnerg Pvt Ltd	Blue Green Algae	• 10-14% increase in crop yield • Highly effective and environment friendly	https://www.algenerg.co.in/blue- green-algae-biofertilizer.htm
TrueSolum	True Algae	*Chlorella*	• Enhanced soil microbial diversity. • More vigorous root systems. • Increased yield. • Better crop quality. • Extended shelf life of their crops	https://truealgae.com/
BYAS-A601	Back of the yard algae sciences	Microalgae	• Potent microalgae based bio-stimulant • Proven natural accelerator of plant growth and phytochemical production. • Improves taste, yield, shelf life • Can be used in controlled environment agriculture and indoor/outdoor fruit and vegetable farming	https://www.algaesciences.com/

Adapted from (Kumar et al., 2022b).

## 9 Concluding remarks

The Use of biofertilizers and natural biostimulants has been on the upward trend in recent years to overcome the dependency on chemical fertilizers and non-renewable resources. Microalgae and cyanobacteria have been proven to enhance and stimulate plant growth and offer systemic immune resistance to various biotic and abiotic stresses. However, the success of microalgae-based biofertilizers and agro-chemical depends on the low cost of biomass production, and lower energy footprints in biomass production technologies. This necessitates the integration of bioremediation and biorefinery models to improve the commercial feasibility of microalgae-based agro technologies. Co-cultivation of microalgae and cyanobacteria in closed-loop hydroponic systems could be an effective system in contributing to global food security. Some key research and developmental directions that are required for the success of microalgae-based technologies for crop productivity enhancement are

- Bio prospection of microalgae and cyanobacterial strains with plant growth promotion properties
- Identification of microalgae strains with wide environmental adaptability such as tolerances to desiccation, wide pH range, temperatures and osmolarity
- Studies evaluating the host plant interaction with microalgae/cyanobacteria strains
- Chemical fingerprinting and establishment of a database on the distribution of plant growth-promoting metabolites such as phytohormones, phenolics and signaling molecules present in microalgae/cyanobacteria strains
- A large number of field trials validating *in vitro* results on fertilizing or stimulatory or pesticidal activity of microalgae-derived extracts
- Development of circular economy loop/routes utilizing agriculture wastewaters and industrial wastes for the cultivation of microalgae biomass and utilizing them in agri-applications
- Safety and risk assessment of biomass produced utilizing wastewaters for heavy metals, xenobiotic residues, horizontal gene transfers
- Development of cost-effective harvesting and dewatering processes for mass production of microalgae biomass
- Education and involvement of farmers on microalgae applications in largescale field trials for product acceptance and widespread use

## Author contributions

VS conceptualized the review. PP, RK and YN created the tables and figures. All the authors contributed in writing the manuscript. VS reviewed the manuscript. All the authors contributed to the article and approved the submitted version.
